# Diversification evidence of bitcoin and gold from wavelet analysis

**DOI:** 10.1186/s40854-023-00495-1

**Published:** 2023-06-01

**Authors:** Rubaiyat Ahsan Bhuiyan, Afzol Husain, Changyong Zhang

**Affiliations:** 1 Faculty of Business, Curtin University, Miri, Malaysia; 2 School of Business, Swinburne University of Technology, Kuching, Sarawak Malaysia

**Keywords:** Bitcoin, Gold, Wavelet, Coherence, Diversification

## Abstract

To measure the diversification capability of Bitcoin, this study employs wavelet analysis to investigate the coherence of Bitcoin price with the equity markets of both the emerging and developed economies, considering the COVID-19 pandemic and the recent Russia-Ukraine war. The results based on the data from January 9, 2014 to May 31, 2022 reveal that compared with gold, Bitcoin consistently provides diversification opportunities with all six representative market indices examined, specifically under the normal market condition. In particular, for short-term horizons, Bitcoin shows favorably low correlation with each index for all years, whereas exception is observed for gold. In addition, diversification between Bitcoin and gold is demonstrated as well, mainly for short-term investments. However, the diversification benefit is conditional for both Bitcoin and gold under the recent pandemic and war crises. The findings remind investors and portfolio managers planning to incorporate Bitcoin into their portfolios as a diversification tool to be aware of the global geopolitical conditions and other uncertainty in considering their investment tools and durations.

## Introduction

Portfolio diversification is a common principle in investment, which is structured with a pool of different investment components with a weak correlation. This allows investors to minimize the level of losses if an unexpected event occurs. With the current advanced technological improvement, constructing an investment technique with minimum risk or maximum opportunity is crucial, as a tiny mistake may drain a significant return within a fraction of time.

Conventionally, gold consistently acts as the principal premium metal. Although the gold price is influenced by the short run variation in the US price level, inflation volatility, credit risk, the USD trade weighted exchange rate (Ghosh et al. 2004; Gorton and Rouwenhorst 2006; Kolluri 1981), besides a considerable parallel relationship between gold and equity returns (Boako et al. 2019), the long run positive relationship between gold and inflation makes it a long-term hedge against inflation (Levin and Wright 2006). Globally, investors use gold as a safe haven for their investment to minimize possible losses (Bassil et al. 2019; Baur and Lucey 2010; Baur and McDermott 2016). This could be owing to its stability irrespective of economic or political circumstances and its hedging ability in times of economic turmoil, e.g., inflation and political crises, as well as the appreciation in its value in the 21st century (Boako and Alagidede 2016; Ghazali et al. 2013; Korkmaz 2018). In addition, investment options in gold are comparatively diverse as well, including gold mining, gold options and futures, gold bullion, and coins (Blose and Shieh 1995; Chua et al. 1990; Jaffe 1989).

Following the global financial crisis in 2008–2009 and the post incidents such as the debt burden of Greece, hyperinflation in the South American countries, and uncertainty created by Brexit, investment strategies with the conventional components are then in question.

Furthermore, owing to the strong correlation between different markets, emerging markets such as Brazil, China, India, Russia, and South Africa are more exposed to shocks flowing from developed economies such as Japan, the United Kingdom, and the United States, and vice versa. The reaction of the stock markets of the latter is hardly predictable, specifically in a crisis. This drives investors to look for alternatives (Mensi et al. 2016).

Right during the global financial crisis emerged Bitcoin (Nakamoto 2009). Despite all the controversies on its validity and risk issues because of its independence from any legal authorities (Baek and Elbeck 2015; Cheung et al. 2015; Cheah and Fry 2015), Bitcoin soon gained its popularity to be considered as a replacement for running bank notes and be compared with the established assets such as gold to test its features (Dwyer 2015; Weber 2016). Only after about thirteen years since its first appearance, it has accounted for a market cap of approximately 900 billion US dollars as of March 31, 2022 (CoinMarketCap 2022).

Although Bitcoin is relatively new in the market, its diversification capabilities have been intensively examined with different combinations (Al-Yahyaee et al. 2018; Bouoiyour and Selmi 2015; Bouri et al. 2017a, b; Ciaian et al. 2016a; Dyhrberg 2016b; Ji et al. 2018; Kristoufek 2013; Yermack 2015). As the most popular global digital currency with a market cap of over 40% as of March 31, 2022 (CoinGecko 2022), Bitcoin has been popularly considered as a new type of asset class (Gangwal 2016; Glaser et al. 2014b; Ram 2019). This could be owing to its unique functioning process that is distinctive to traditional currencies (Bouri et al. 2018; Xu et al. 2019). For instance, it does not require any channel for making transactions. Particularly, owing to its weak correlation with traditional assets, its role as a valuable diversifier has been progressively highlighted (Firth et al. 2019; Phillips and Gorse 2018; Platanakis and Urquhart 2020), although possible caution remains that investors in stock indices including S&P 500 should keep a close eye on the unstable market trend of Bitcoin (Erdas and Caglar 2018). Additionally, it is shown that gold, the conventional safe heaven, may be replaced by Bitcoin in an investment portfolio (Henriques and Sadorsky 2018), and Bitcoin allows for facing the modern environmental challenges better than gold owing to its significantly small social and economic impact (Cocco et al. 2019b).

As Bitcoin gains increasing attention from investors as an investment asset for portfolio diversification, it is essential and critical to test its similarity in return properties in different markets (Ciaian et al. 2016b; Hong 2017). In addition, this study is motivated by four factors: the sudden detection and worldwide spread of COVID-19 in early 2020, the downward movement of the global stock markets to the lowest following the loss of market confidence, the steep increase of Bitcoin price following the announcement of the COVID-19 pandemic in early 2020 and an abrupt decrease at the end of 2021, and the Russia-Ukraine war in early 2022. All these factors remind the importance of testing the time-varying diversification capability of Bitcoin against stock markets during both normal and uncertain periods.

The price of gold increases when more uncertainty in the market is announced. This fact is well established and is not new in the market. The steep movement of the Bitcoin price brings the necessity of a combined look of both Bitcoin and gold with both emerging and developed markets, which is still lacking in the literature. Therefore, this study compares Bitcoin and gold returns against six representative market indices: FTSE 100, FTSE Indonesia, FTSE Bursa Malaysia KLCI, NIKKEI, NIFTY, and S&P 500, as well as Bitcoin return with gold return. Additionally, this study considers two markets in the ASEAN, which is widely considered one of the fastest growing regions in the coming years.

This study aims to answer the question whether Bitcoin provides the same diversification benefit as gold for global investors in different markets. The study contributes to the literature by empirically measuring the connectedness of Bitcoin and gold, testing the volatility of different stock markets in different crises along with Bitcoin and gold, and evaluating how the correlation factors vary during crises.

Through the wavelet and multivariate generalized auto-regressive conditional heteroskedasticity (GARCH) methods, which have been proven powerful tools in economics and finance (Agyei et al. 2022; Bhuiyan et al. 2019; Bilgili et al. 2020; Goodell and Goutte 2021; Guesmi et al. 2019; Kuşkaya and Bilgili 2020; Qiao et al. 2020; Shehzad et al. 2021a, b), it is shown that both Bitcoin and gold were positively correlated during the COVID-19 pandemic and negatively correlated during the recent Russia-Ukraine war. It is also observed that although the stock markets were volatile at the start of the COVID-19 crisis, they stabilized thereafter. The volatility of the stock market and gold was significantly lower than that of Bitcoin. In addition, it is revealed that the correlation between Bitcoin and gold was mixed throughout the two crises. That is, for some markets, the correlation was positive during the COVID-19 pandemic and negative during the Russia-Ukraine war, whereas some other markets experienced upward correlation trend during the first crisis and downward correlation trend during the second crisis. The findings are complementarily verified by examining the time-varying hedge ratios.

Bitcoin can provide diversification benefit to global investors during crises. Additionally, Bitcoin is different from gold in offering diversification benefit to its users. In terms of diversification ability during crises, Bitcoin outperforms gold. The findings have valuable implications for investors, portfolio managers, and policy makers in safeguarding wealth.

Related existing studies are discussed in "Literature review" section. In "Methodology" section, the continuous wavelet transform (CWT) and multivariate GARCH are described. The data used to test the correlation and the results are presented in "Results" section. "Conclusion" section concludes the study with a brief discussion on possible implications.

## Literature review

In this section, existing studies on possible diversification evidence of gold and Bitcoin are further reviewed, complementary to those discussed in "Introduction" section.

### Gold as a diversifier

Owing to its risk reduction ability, inclusion of gold as a stabilizer for a portfolio has been well established (Ciner 2001). Investors commonly turn to gold to protect their investment during crises while keep a certain portion of gold within the portfolio to balance their portfolio and safeguard an unexpected event during normal periods.

Conventionally, typical portfolio components include gold, stock, bond, and commodity. Among them, stock and bond tend to replicate each other (Baur 2010; Cascino 2017; Fang et al. 2017; Panchenko and Wu 2009). Gold features negative correlation with those assets (Chkili 2016; Shahzad et al. 2017; Ziaei 2012). Therefore, gold is considered a valuable diversifier for portfolio risk.

For its diversification ability, gold is a favorable diversifier for a portfolio. It reduces the downside risks and boosts the portfolio return simultaneously (Azar 2016; Bouri et al. 2020b; Matos and Evans 2017; Pho et al. 2021).

Additionally, the GARCH model based on 28 years data starting from January 1, 1976 demonstrates that the three mined metals, including gold, silver, and platinum, act as diversifiers for the S&P 500 and improve the portfolio performance by a significant margin compared with the ones without them (Hillier et al. 2006).

### Bitcoin as a diversifier

As a leading cryptocurrency, Bitcoin has managed to make room among the elite assets in just over a decade since its first entry (Gangwal 2016; Glaser et al. 2014a; Ram 2019).

Initially, Bitcoin was claimed more as an asset rather than a mere currency (Baek and Elbeck 2015; Dyhrberg 2016b; Glaser et al. 2014b). Gradually, it successfully found its way to take over the use of paper bills, as a number of multinational companies such as Airbnb, Amazon, Dell, and Starbucks have started to accept payments through Bitcoin (Dyhrberg 2016a; Yermack 2015). However, it still sometimes fails to perform all the attributes that conventional currencies do though (Gervais et al. 2014).

At the initial stage, more focus was on the legality of Bitcoin (Barber et al. 2012; Tu and Meredith 2015). Later, it was progressively considered as an investment asset and became a point of interest in terms of investment owing to its healthy return structure (Dyhrberg et al. 2018; Koutmos 2018; Nadarajah and Chu 2017). It has shown to act as a better diversifier in a portfolio owing to its exceptional low correlation with other assets (Baur et al. 2018b; Bouri et al. 2017b; Chen et al. 2020; Guesmi et al. 2019).

To this extend, Bitcoin is a valuable diversifier against conventional assets (Bouri et al. 2017b). Similarly, it is revealed that Bitcoin provides a healthy return when included in a conventional bond and stock portfolio (Brière et al. 2015; Platanakis and Urquhart 2020). When used in global industry portfolio and bond index diversification, Bitcoin is shown to be a valuable tool to minimize the risk (Akhtaruzzaman et al. 2020). It is also recommended to use Bitcoin instead of conventional assets as a diversification tool for minimizing liquidity risk (Ghabri et al. 2021). Although Bitcoin may not be the same as gold within a portfolio (Cocco et al. 2019a; Klein et al. 2018), it is evidenced that Bitcoin can replace gold (Bouri et al. 2018; Henriques and Sadorsky 2018; Hong 2017).

In addition, examining Bitcoin as a diversifier under different market conditions, evidence is indicated in favor of Bitcoin as a potential diversifier in most cases for most assets in a conventional portfolio, given that the market stays normal (Bakry et al. 2021). It also suggests that risk-taking investors should include Bitcoin while risk-averse investors should stay caution of the price movement. Similar finding is noted that Bitcoin is suitable for risk-taking investors while gold is suitable for risk-averse investors (Pho et al. 2021).

Recent studies established that inclusion of other cryptocurrencies within a well-diversified portfolio could boost the portfolio return, whereas Bitcoin plays the majority of the role (Boiko et al. 2021; Wang and Ngene 2020). Bitcoin acts as a favorable portfolio diversifier owing to its lower connectedness with most of the conventional financial assets (Brière et al. 2015; Bouri et al. 2020b; Eisl et al. 2015; Ghorbel and Jeribi 2021a, b; Wu et al. 2014), Wa̧torek 2021. Bitcoin contributes significantly to a well-diversified portfolio despite some flaws such as volatility (Jeribi et al. 2022).

To assess whether Bitcoin can be the alternative for gold, stocks, and currencies, the relationship between Bitcoin and traditional assets is examined in the tail sense (Chen et al. 2001; Kalyvas et al. 2020). It is asserted to be a good diversification asset for stocks because its left tail is uncorrelated with those of stock indices of developed economies such as Euro Stoxx 50, Nikkei 225, and S&P 500 (Feng et al. 2018). It is complementarily reported that Bitcoin is a safe haven for the U.S. equity indices (Bouri et al. 2020a). It is alternatively shown that Bitcoin cannot be a safe-haven property for stock market investment, specifically during extreme market periods (Hussain Shahzad et al. 2019).

### Bitcoin and gold

In most cases, Bitcoin is put aside of gold due to their shared properties. Neither Bitcoin nor gold is bordered or governed by any national authorities. Both need to be mined and are priced independently. Although both can be used to trade, the price formation pattern of Bitcoin is rather unique. The price does not correlate with those of others and is determined through the forces of demand and supply, irrespective of any economic or global events (Bouoiyour et al. 2015; Dastgir et al. 2019; Kristoufek 2015; Polasik et al. 2015).

Gold has been a safe haven for investment for a long time owing to its low correlation with other assets, specifically during market turmoils such as inflation, financial, and political crises (Balcilar et al. 2017a, b; Baur and McDermott 2010; Baur 2010; Boako and Alagidede 2016; Nguyen et al. 2016; Troster et al. 2019). However, Bitcoin manages to find a position alongside other investment assets within the portfolio and it is largely considered as an alternative to gold. Hence, it is named as the digital gold (Popper 2015).

The comovement in price changes of Bitcoin and gold is evidenced based on the data from 2010 to 2015 (Dyhrberg 2016b). It is also claimed that Bitcoin and gold show unique movement patterns, making them clearly distinguishable (Baur et al. 2018a). In examining the reaction of the Bitcoin price return to variation in the gold price return, U.S. stock market return, interest rates, and crude oil price using over eight years data starting from 2010, it was established through the NARDL method that both Bitcoin and gold price returns are statistically significant and positive (Jareño et al. 2020). In testing the diversification capabilities of gold and Bitcoin against G7 stock markets, evidence suggests the distinctive characteristics between the two and Bitcoin offers a weaker diversification benefit participant of the G7 markets (Shahzad et al. 2020).

Investors are keen to search for a perfect diversifier. The positive diversification benefit from using Bitcoin has taken a new turn since the start of the COVID-19 pandemic. It is clear that although Bitcoin can barely minimize the portfolio risk, it can boost the return (Hasan et al. 2021). Comparing the diversification ability of Bitcoin against gold for the China market during the first few months of the COVID-19 pandemic to April 30, 2020, it is revealed that Bitcoin boosts the portfolio return while increasing the risk level simultaneously, whereas inclusion of gold in the portfolio eliminates the risk by a significant margin compared with Bitcoin (Pho et al. 2021). Hence, gold is concluded as the better diversifier for Chinese portfolios. In contrast, from the study of the Bitcoin price movement because of changes in the prices of precious metals such as gold and silver in the U.S. market, Bitcoin follows the trend of gold in almost all the cases observed (Agnese and Thoss 2021).

Bitcoin is somehow in parallel labeled as a bubble in the financial markets, as its price formation focuses on technical information and speculation (Ante and Fiedler 2021; Bouoiyour et al. 2015; Celeste et al. 2020). As gold futures also sometime feature a bubble nature, from hedging the bubble behavior of Bitcoin using the gold futures, it is hinted the distinctive characteristics of Bitcoin, compared with gold futures in terms of volatility as well as causality (Kang et al. 2019). However, the highest level of correlation between the pair is mainly during 2012–2015 only.

For volatility, Bitcoin enhances portfolio diversification as its spillover effect is at minimum, as compared with other financial assets (Burnie 2018; Guesmi et al. 2019). In a similar manner, a negative correlation is stated between the volatility of Bitcoin and that of other assets. Additionally, it is shown that the volatility of Bitcoin decreases during financial crises, indicating its ability to act as a favorable diversifier (Conrad et al. 2018). In another study, it was established that Bitcoin is highly volatile, undermining most diversification opportunities, whereas its weaker correlation with gold and oil makes it work as a diversifier for a long-term horizon (Ozturk 2020).

## Methodology

The time-varying and time-scale-dependent returns comovements between the sample indices are assessed using a CWT. Specifically, given the original time series as a function of time, the CWT recasts it separately into a function of two variables: time and frequency. The series correlation in a two-dimensional diagram helps to identify and interpret the pattern or hidden information. The analysis of correlation between two CWTs is known as the wavelet coherence. The diagram specifies the degree of correlation between two variables with varying time and frequency. Hence, the wavelet method provides an alternative representation of the variability and relationship structure of certain stochastic processes on a scale-by-scale basis.

Particularly, stylized facts such as nonstationary or nonlinear lead-lag interactions are commonly observed in financial time series partially due to heterogeneous expectations and risk perceptions of investors across varying investment horizons. In this case, most traditional econometric techniques may not be applied directly and the wavelet method readily steps in.

To measure the dynamic conditional correlation (DCC) of a portfolio, the multivariate DCC-GARCH approach is applied. In particular, for risk assessment concerning the tail properties of the return distributions to identify diversification benefits, the DCC model with a multivariate *t*-distribution captures the fat-tailed nature of asset returns.

### Continuous wavelet transform

To maintain consistency with the suggestion that a balance should be retained between the sample size and the length of the wavelet filter (In and Kim 2013), this study adopts the least asymmetric wavelet filter of length L = 8, represented by LA (8), based on eight non-zero coefficients. It is revealed by studies on high-frequency data that a moderate-length filter such as L = 8 is suitable for dealing with the characteristics or features of time series data (Gençay et al. 2001, 2002; Percival and Walden 2000). It is claimed that an LA (8) filter provides smoother wavelet coefficient than other filters such as the Haar wavelet filter.

Let $\psi \in L^2(\mathbb {R})$, the set of square integrable functions, be a mother wavelet, satisfying the admissibility condition practically equivalent to

$$\begin{aligned} \int _{-\infty }^{\infty } \psi (t) dt = 0 \end{aligned}$$

for recovering the time series from its wavelet transform (Daubechies 1992). The CWT $W_x(s, \tau )$ with respect to $\psi$ is obtained by projecting the examined time series $x(t) \in L^2(\mathbb {R})$ onto $\psi$. That is,

$$\begin{aligned} W_x(s, \tau ) = \int _{-\infty }^{\infty } \frac{1}{\sqrt{s}} \psi (\frac{t - \tau }{s}) x(t) dt, \end{aligned}$$

where *s* is a scaling or dilating factor that controls the length of the wavelet or the position of wavelet in the frequency domain, and the location parameter $\tau$ indicates where the wavelet is centered or the position in the time domain. Hence, the wavelet transform provides information concurrently on time and frequency by mapping the original series into a function of *s* and $\tau$.

To study the interaction between two time series *x* and *y* or how closely they are integrated by linear transformation, the wavelet coherence is applied. That is,

$$\begin{aligned} R^2(s, \tau ) = \frac{|S\big (s^{-1} W_{xy}(s, \tau )\big )|^2}{S\big (s^{-1} |W_x(s, \tau )|^2\big ) S\big (s^{-1} |W_y(s, \tau )|^2\big )}, \end{aligned}$$

where *S* is a smoothing operator that can be written as a convolution in time and scale, *s* is a wavelet scale, $W_x(s, \tau )$ is the wavelet transform of *x*, $W_y(s, \tau )$ is that of *y*, and $W_{xy}(s, \tau ) = W_x(s, \tau ) W_y^*(s, \tau )$ is the cross wavelet transform of the two time series (Aguiar-Conraria et al. 2008; Madaleno and Pinho 2012; Vacha and Barunik 2012).

The wavelet squared coherence $R^2(s, \tau ) \in [0, 1]$ measures the comovement correlation between the time series. A value close to zero indicates weak correlation and a value close to one implies strong correlation.

### Multivariate GARCH

When the multivariate GARCH model is adopted, the estimation of the DCC method involves two steps.

In the first step, the univariate volatility parameters are measured for each variable. There are two GARCH equations estimated for two variables. For instance, in the asymmetric GARCH equation (Glosten et al. 1993),

$$\begin{aligned} h_t = b_0 + b_1 h_{t-1} + c_1 \varepsilon _t^2 + c_2 \varepsilon _t^2 I_{\{\varepsilon _t > 0\}}, \end{aligned}$$

where *I* is an indicator function that is equivalent to 1 if the standardized residual of the series $\varepsilon = \{\varepsilon _t\}$ is positive and equivalent to 0 otherwise. A negative value of $c_2$ implies that periods of higher variances follow more immediately periods of negative residuals compared with periods of positive residuals. To estimate the residual, the GARCH equation is measured for each variable. Similarly, a multivariate GARCH model can be expressed as follows (Bauwens et al. 2006):

$$\begin{aligned} h_{i, t} = b_{i, 0} + \sum _{i=0}^n b_{i, j} h_{j, t-1} + \varepsilon _{i, t}, i = 1, \dots , n. \end{aligned}$$

Next, the residuals resulting from the first stage are taken as inputs to estimate a time-varying correlation matrix by measuring the DCC equation parameters,

$$\begin{aligned} H_t = D_t R_t D_t, \end{aligned}$$

where $H_t$ is the conditional covariance matrix, $D_t$ is the diagonal matrix of the conditional time-varying standardized residual that is acquired from the univariate GARCH models as the on-diagonal elements or variance, and $R_t$ is the time-varying correlation matrix as the off-diagonal elements (Engle 2002; Tse and Tsui 2002).

The likelihood of the DCC estimator is then

$$\begin{aligned} L = -\frac{1}{2} \sum _{t=1}^T (n \log {2\pi } + 2 \log {|D_t|} + \log {|R_t|} + \varepsilon _t^{\prime } R_t^{-1} \varepsilon _t). \end{aligned}$$

Volatility component $D_t$ is maximized in the first step. That is, the log likelihood is reduced to the sum of that of the univariate GARCH equations.

Depending on the estimated $D_t$, correlation component $R_t$ is maximized in the second step, with elements $\varepsilon _t$ being obtained from the first step. The nonnegative parameters $\alpha$ and $\beta$ satisfying $\alpha + \beta \le 1$ in the following DCC equation are then evaluated.

$$\begin{aligned} R_t = (1 - \alpha - \beta ) R + \alpha \varepsilon _{t-1} \varepsilon _{t-1}^{\prime } + \beta R_{t-1}. \end{aligned}$$

Hence, $R_t$ is the weighted average of three matrices. A strong degree of persistence is indicated in the series for correlation $R_t$ if $\beta$ is close to 1, whereas high persistence is suggested in the conditional variance if $\alpha + \beta$ is close to 1. For both the conditional correlations and variances, the model has the GARCH-type dynamics. The time-varying conditional variances measure the uncertainty that provides insight into the causes of movement in the variance.

If $\alpha = \beta = 0$, $R_t$ is simply the time-invariant *R* with unit diagonal elements and the constant conditional correlations (CCC) model, a univariate GARCH process followed by the conditional variance for each return (Bollerslev 1990), is sufficient. Specifically,

$$\begin{aligned} h_{it} = \omega _t + \sum _{j=1}^p \alpha _{ij} \varepsilon _{i, t-j}^2 + \sum _{j=1}^q \beta _{ij} h_{i, t-j}, \end{aligned}$$

where $\alpha _{ij}$ represents the ARCH impact on or short-term persistence of shocks to return *j* and $\beta _{ij}$ reflects the GARCH effects of shocks on return *i* to long term persistence. Hence, the CCC model assumes independence of conditional variances across returns and does not support asymmetric behavior.

## Results

To investigate the interrelation between the asset classes, the sample data are first described in the Data section and the findings are presented in the Findings section.

### Data

The Bitcoin and gold prices, as well as six indices are considered in this study: FTSE 100, FTSEIndo for FTSE Indonesia, FTSEMY for FTSE Bursa Malaysia KLCI, Nikkei 225, Nifty 50, and S&P 500.

The Financial Times Stock Exchange 100 Index is a capitalization-weighted index of the 100 most highly capitalized companies traded on the London Stock Exchange. The FTSE Bursa Malaysia KLCI index comprises of the largest 30 companies by full market capitalization on the main board of Bursa Malaysia. The Nikkei 225 stock average is a price-weighted average of 225 top-rated Japanese companies listed in the first section of the Tokyo Stock Exchange. The Nifty 50 is the flagship index on the National Stock Exchange (NSE) of India, computed by adopting a float-adjusted and market capitalization weighted methodology. The index tracks the behavior of a portfolio of blue-chip companies, the largest and most liquid Indian securities domiciled in India and listed on the NSE. The S&P 500, a stock market index that measures the stock performance of 500 large companies listed on stock exchanges in the United States, is widely regarded as the best single gauge of the large-cap U.S. equities and serves as the foundation for a broad range of investment products.

The sample data are collected for the period from January 7, 2014 to May 31, 2022, which matches the availability of the Bitcoin data and covers both the pre-pandemic and post-pandemic periods. To make it consistent with the indices, only the Bitcion prices on weekdays are used. For each of the six indices, the value of the previous trading day is taken by convention in case the market is closed for a trading day in the other markets. The indices along with the Bitcoin and gold prices are transformed to market returns through the natural logarithmic difference. The respective sources and tickers of the data are summarized in Table 1.

**Table 1:** Specification of data

Index	Source	Ticker
Bitcoin price	CoinMarketCap	BITCOIN
Gold spot price (Dollars per Ounce)	USAGOLD	GOLD
Financial times stock exchange 100 index	Investing.com	FTSE 100
FTSE Indonesia	Investing.com	FTSEINDO
FTSE Bursa Malaysia KLCI	Investing.com	FTSEMY
Nikkei 225	Investing.com	NIKKEI
NSE Nifty 50	Investing.com	NIFTY
Standard & Poor’s 500	Investing.com	S&P 500

The corresponding descriptive statistics are presented in Table 2, where 2183 daily observations are considered. Notably, Bitcoin has the highest mean daily return of $0.1579\%$ compared with the second highest $0.0451\%$ of NIFTY, and it is the most volatile with a daily variation of $6.0427\%$ compared with the second highest $1.3300\%$ of FTSE Indonesia.

**Table 2:** Descriptive statistics of data

	Mean	Median	Min	Max	St. dev.	Skewness	Kurtosis
BITCOIN	0.00157884	0.00168350	$-$0.84882867	1.47417980	0.06042700	5.00913968	183.97572597
GOLD	0.00018085	0.00020232	$-$0.05264572	0.05133427	0.00878963	$-$0.16948420	3.82679525
FTSE 100	0.00005610	0.00035512	$-$0.11512428	0.08666807	0.01019099	$-$0.90140378	13.63310120
FTSEINDO	0.00022323	0.00000000	$-$0.08642203	0.14354692	0.01330001	0.15244163	11.70568152
FTSEMY	-0.00006996	0.00000000	$-$0.05404726	0.06626284	0.00669411	$-$0.24011278	9.85197689
NIKKEI	0.00024703	0.00023621	$-$0.08252932	0.07731370	0.01246332	$-$0.14256030	5.11314985
NIFTY	0.00045135	0.00030058	$-$0.13903754	0.06414547	0.01052545	$-$1.60142040	20.76484810
S&P 500	0.00037536	0.00050300	$-$0.12765214	0.08968316	0.01098850	$-$0.98308081	19.77723529

### Findings

Wavelet analysis, which is conducted through MATLAB in this study, reveals if the comovement is low or high over time and across frequency, catching conceivable varying features in the relationship between the time series with respect to both time and frequency.

The coherence is displayed from Figs. 1, 2, 3, 4, 5, 6, 7, 8, 9, 10, 11, 12 and 13, in the Wavelet coherence section.

In each figure, the horizontal axis represents the time interval and the vertical axis denotes the scale. The frequency has an inverse relationship with the scale. For the ease of interpretation and understanding, the frequency is converted into number of days, to reflect investment horizons in short term, medium term, and long term (Baruník and Křehlík 2018). To synchronize with the wavelet coherence representation, the investment horizons that double subsequently starting from two days are defined accordingly with 2–16 days as short-term, 16–64 days as medium-term, and 64–256 days as long-term horizons. Hence, the wavelet transformation contributes to the analyzing of portfolio diversification opportunities in different investment horizons.

Blue indicates a low correlation or weak linkage between the two time series and yellow indicates a high correlation or strong interdependence between them. The area where the comovement is statistically significant is separated by a dark thick line at the significance level of 5%. Outside the contour where the inference is reliable is the cone of influence shown as a lighter shade, where edge effects might distort the illustration (Grinsted et al. 2004; Torrence and Compo 1998). In addition, the relative phase relationship is denoted by arrows, with in-phase pointing right and anti-phase pointing left.

The conditional volatilities and correlations are illustrated from Figs. 14, 15, 16 and 17 in the Conditional volatility and correlation section.

#### Wavelet coherence

**Fig. 1:**
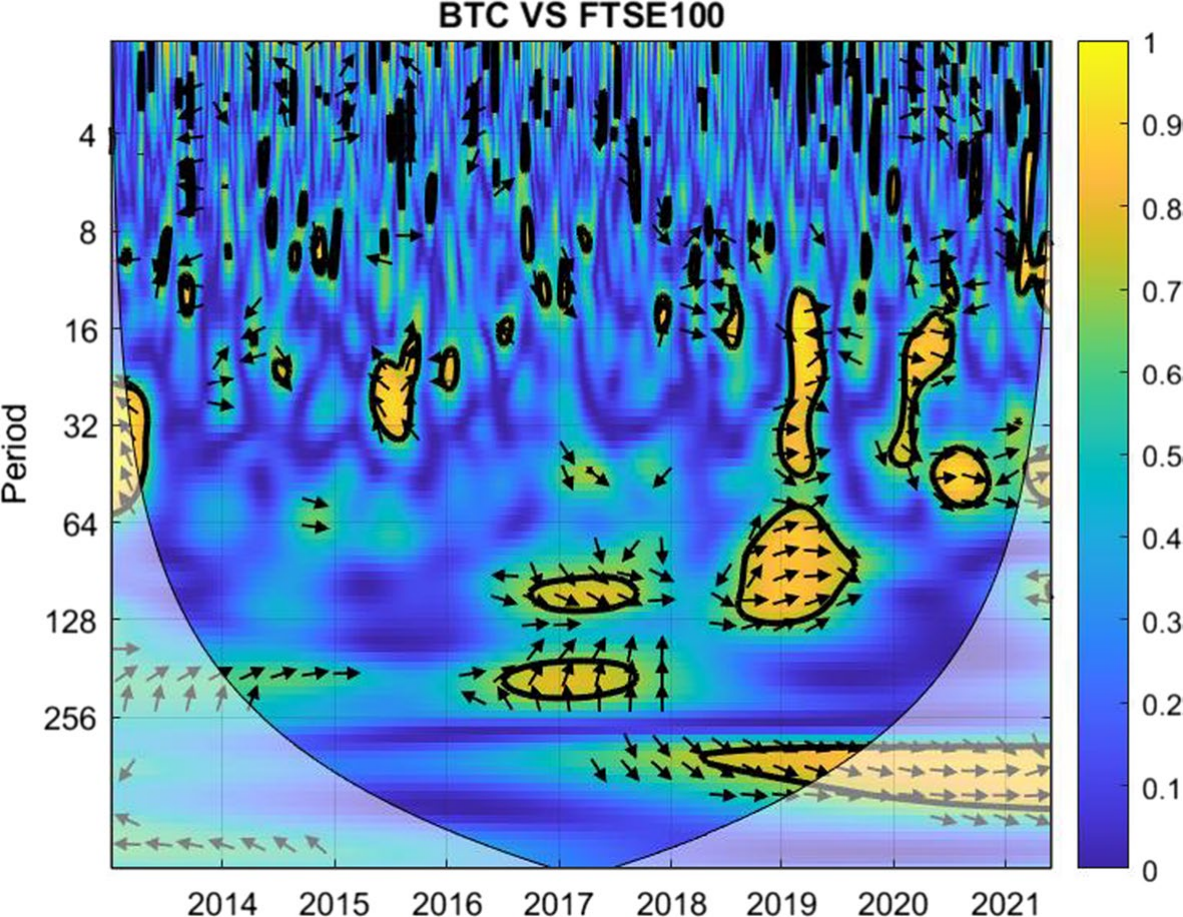
Wavelet coherence between Bitcoin and FTSE 100

As shown in Fig. 1, blue and light blue dominate for short-term horizons of 2–16 days, which indicates that Bitcoin is not strongly correlated with FTSE 100. Hence, it offers portfolio diversification for the time period examined.

For medium-term investment horizons of 16–64 days, similar diversification opportunities are obtained, except for late 2016, early 2020, and early 2021, which witnessed relatively high correlation between the two time series. The two yellow marks during the early quarters of 2020 and 2021 could be attributed to the sudden shocks of the first pandemic announcement and the second wave announcement, respectively.

For long-term investment with holding periods of 64–256 days, low comovement in return occurs between Bitcoin and FTSE 100, favorable for portfolio diversification, particularly for the first three years. There are variations in the following years. The possible interpretation of the relatively high correlation between the pair in 2017 and 2018 could be attributed to the rise of uncertainty with respect to the Brexit issue in the U.K. and the US-China trade war, whereas the high correlation in 2019 and 2020 could be directly related to the COVID-19 pandemic.

Overall, it exhibits most blue regions in the short-term horizons, more green regions in the medium-term horizons, and a mix in the long-term horizons. The analysis suggests that investors should use Bitcoin to diversify against FTSE 100, irrespective of the market condition, more in the case of considering short-term investment plans. In particular, the green and yellow marks in 2021 and 2022 indicate closer comovement between the pair throughout both the crisis and post-crisis or recovery periods, which dims the chance for diversification evidence of Bitcoin against FTSE 100 during the crisis and the immediate post-crisis periods.

**Fig. 2:**
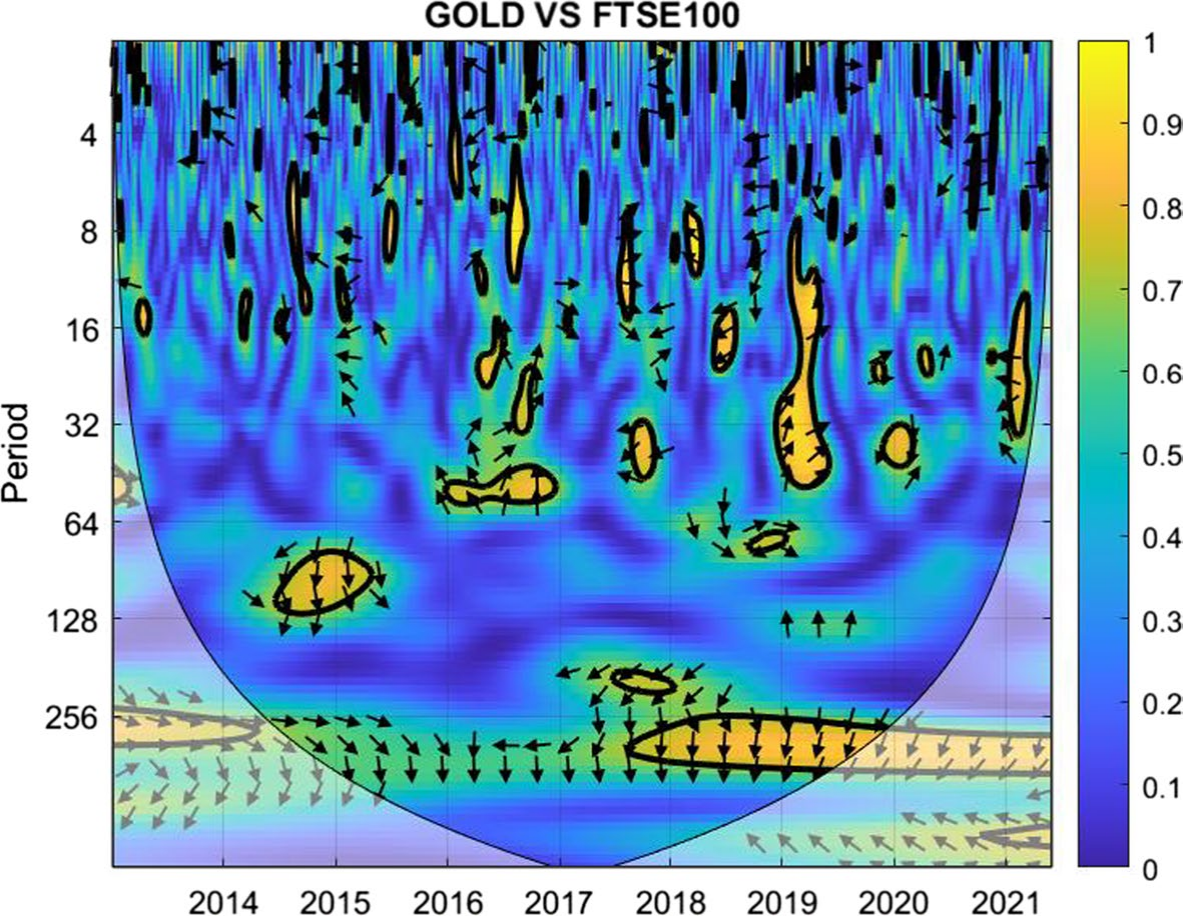
Wavelet coherence between gold and FTSE 100

Between gold and FTSE 100, Fig. 2 suggests short-term diversification opportunities in all the years after possible moderate adjustments at a few negligible light green and yellow marks. For medium-term investors, diversification opportunities were present in most years, except for 2017, early 2020, and early 2022. The yellow marks indicate comparatively high correlation between the pair during crises.

For long-term investment plans, diversification consistently exists for investors in all years except for 2015, in the year of which the yellow mark could be attributed to the Chinese market crash. Concerning the other crises in relation to FTSE 100, including the Brexit and the US-China trade war in 2017 and 2018, blue to light green indicate suitable diversification opportunities. Moreover, the dominating blue region shows that the correlation between gold and FTSE 100 remains weak during the COVID-19 pandemic.

Overall, this combination offers the best diversification opportunities for short-term investors. Meanwhile, for investors aiming for long-term horizons, gold may help to diversify the market risk of FTSE 100 as well, particularly given that the financial market condition is either stable or in the recovery phase.

**Fig. 3:**
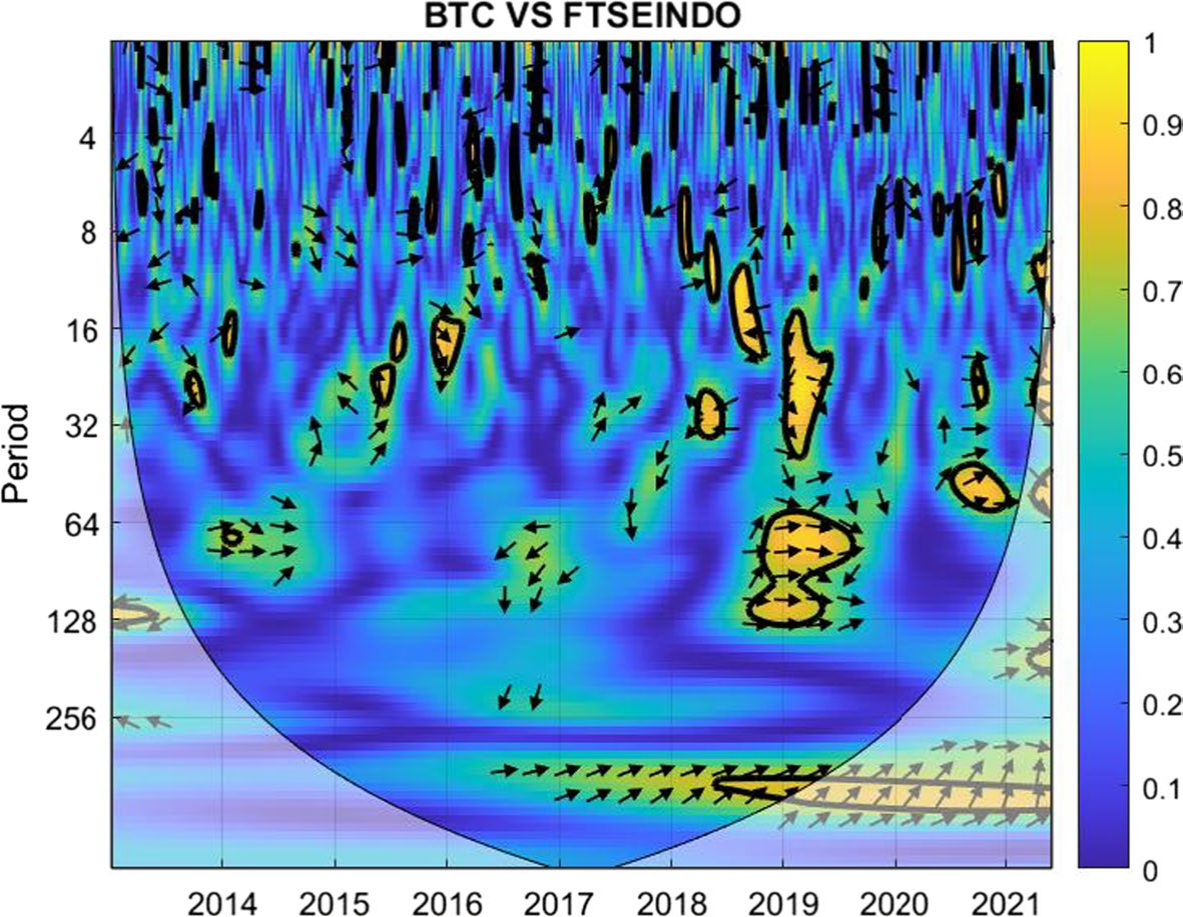
Wavelet coherence between Bitcoin and FTSEINDO

The insignificant yellow marks in Fig. 3 show that the correlation between Bitcoin and FTSEINDO is weak for different horizons in almost all years, providing clear options for diversifying, particularly for short-term investment horizons. The main visible exceptions are the early days of the COVID-19 pandemic for certain medium-term and long-term investment periods.

The blue and green colors during most peak periods of the crisis related to the COVID-19 pandemic suggest that Bitcoin may be a suitable diversifier for FTSE Indonesian investors in all investment horizons, given that the investors are relatively patient and hold their investment without panicking during the early days of the crisis.

**Fig. 4:**
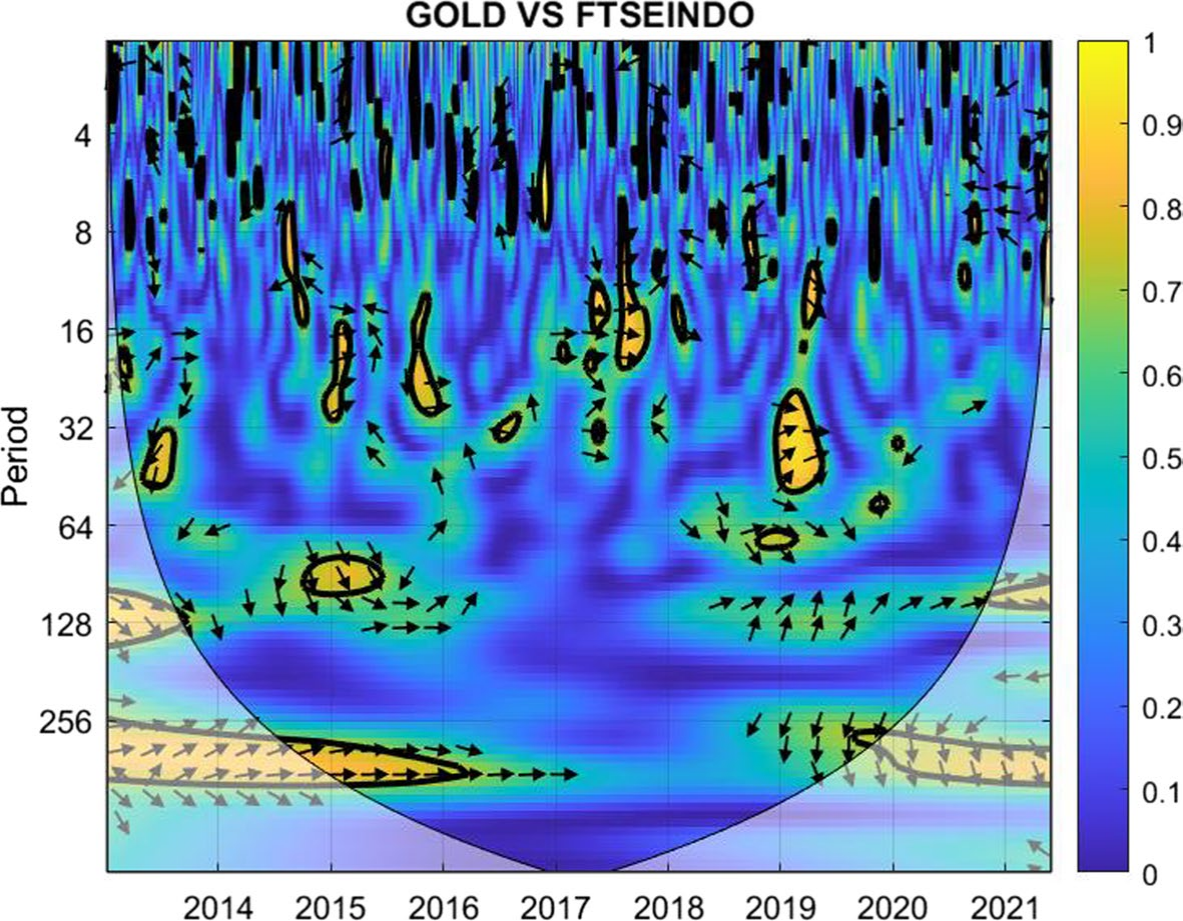
Wavelet coherence between Gold and FTSEINDO

For short-term investment, Fig. 4 shows nearly identical representation for gold with FTSEINDO as in the case of Bitcoin. For medium-term and long-term horizons, it appears somehow different.

The yellow marks across the years indicate higher connectedness between the two for medium-term investment. Surprisingly, the correlation becomes weak during the post-COVID announcement periods. For long-term investment, the correlation stands relatively high for the horizon of 64–128 days, contrary for investment plans designed around 128–256 days. Investors could benefit from the weak correlation between gold and FTSE Indonesia in the longer term.

Hence, gold is a suitable diversifier against the FTSE Indonesian risk mainly for short-term investment. For medium-term and long-term investment, gold may be used during the normal times and not during crises. However, for long-term investors with investment plans over 128 days, gold may be considered as a diversifier for FTSE Indonesia.

**Fig. 5:**
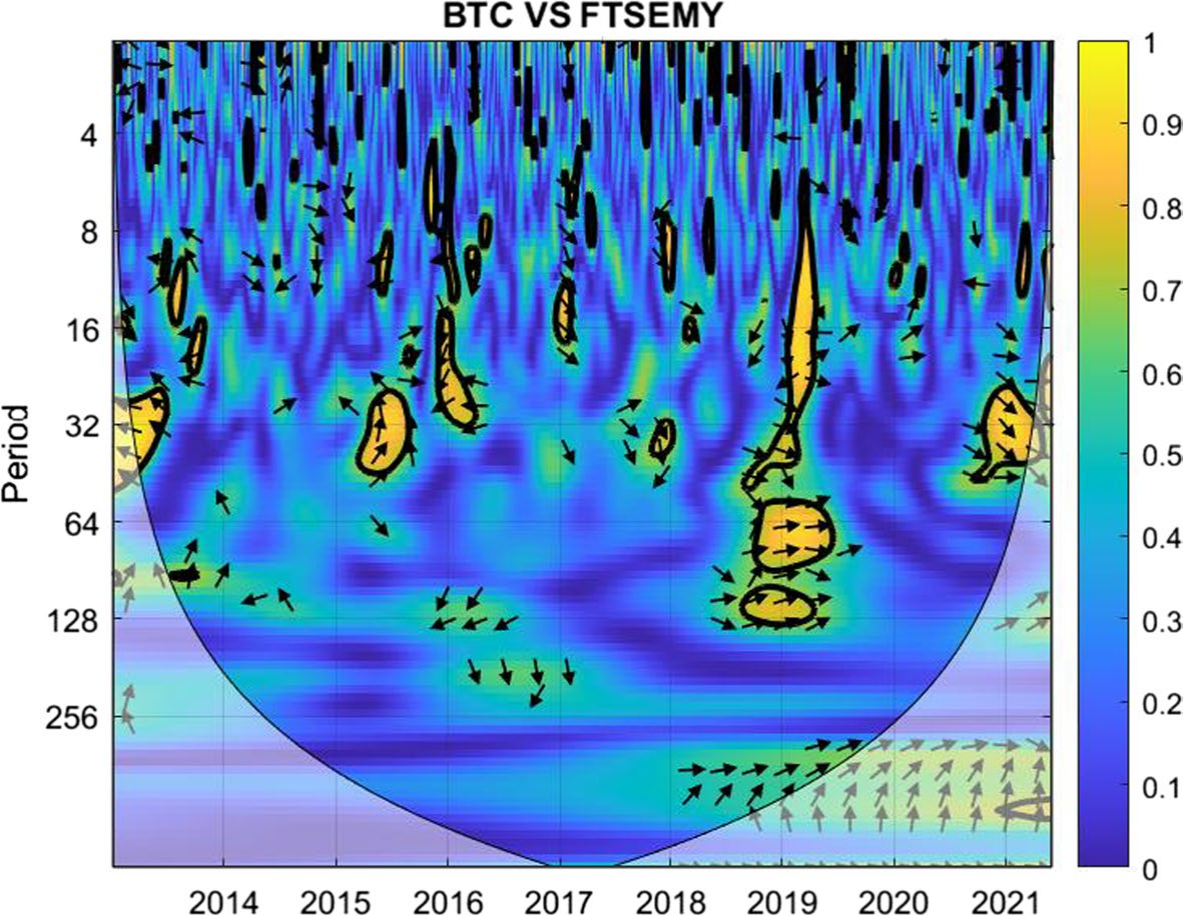
Wavelet coherence between Bitcoin and FTSEMY

For the combination of Bitcoin and FTSEMY, Fig. 5 shows notable areas with green to light blue compared with deep blue, which may indicate relatively low diversification opportunities, particularly for investment horizons beyond eight days.

The yellow and green regions during 2014 are consistent with the period when the Malaysian economy was facing the thread of the nose-diving global energy prices. The pair then experienced higher correlation in 2016 and early 2017, which could have been owing to the slacking GDP resulting from the shrinking net export of the economy, with a fall in export to its largest exporting counterparts such as China, Japan, and the Europe. The pair again observed high correlation for nearly one year starting from the second half of 2019 and in late 2021 as well, mainly owing to the COVID-19 pandemic. The color then turns back to deep blue with the nation moving away from the pandemic to an endemic, hinting diversification opportunities for long-term investment horizons.

The analysis suggests that investors may use Bitcoin as a diversifier for FTSE Malaysia without worrying for short-term investment. When planning investments for longer horizons, investors are urged to look for economic conditions of the nation in deciding their diversification tools. For post-crisis periods when the correlation factors seem to weaken over time, Bitcoin may be a choice worth considering for investors with a long-term investment plan.

**Fig. 6:**
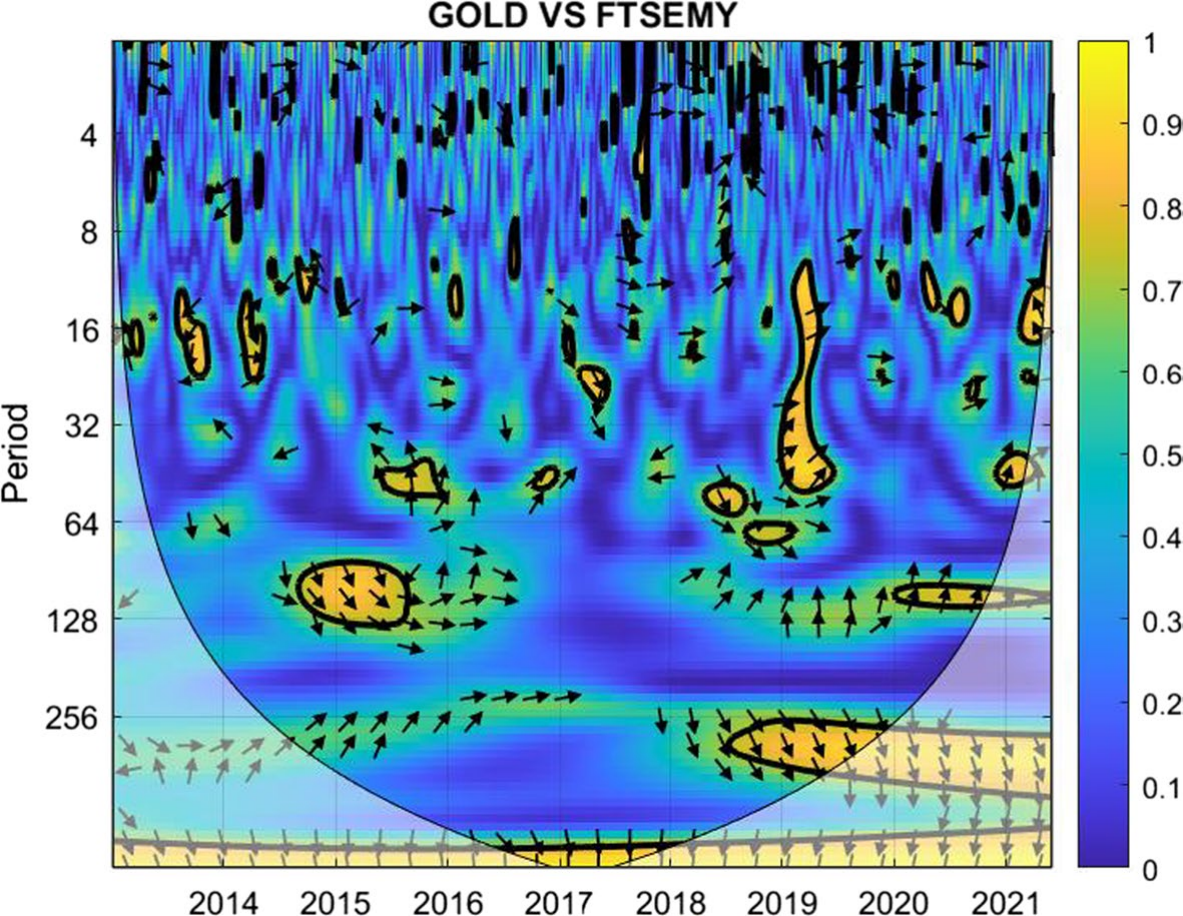
Wavelet coherence between gold and FTSEMY

Figure 6 shows the possibility of gold as a mild diversifier for Malaysian investors up to investment horizons of eight days, in line with the case of Bitcoin. Beyond that, the more non-blue regions dim the diversification possibilities.

Green and yellow marks are clearly spotted in 2015 to 2017 when the Malaysian economy experienced the decline in export and GDP alongside the additional external tensions owing to the Brexit issue in Europe. Similar marks are noted in 2019 and the subsequent years when the world economy was hit by the global pandemic and the Russia-Ukraine war that followed up immediately.

The wavelet coherence indicates the ineffectiveness of using gold to diversify risks associated with the FTSE Malaysia index during crises, whereas gold can be considered as a suitable diversifier for the index in all investment horizons during a normal market condition.

**Fig. 7:**
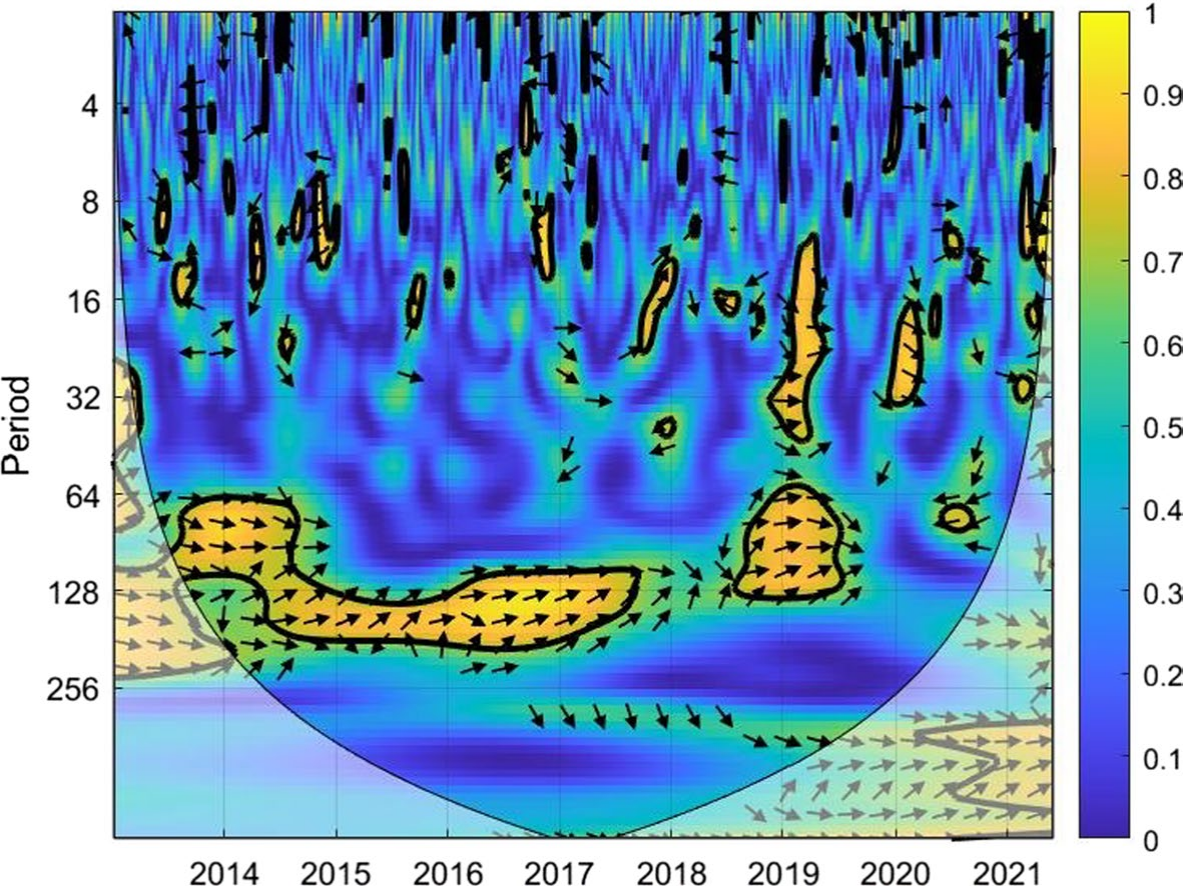
Wavelet coherence between Bitcoin and NIKKEI

The widely spreading green and yellow regions in Fig. 7 denote less beneficial to diversify owing to high correlation between Bitcoin and Nikkei, particularly for medium-term and long-term investment horizons.

During the last quarter of 2013, the Japanese government introduced a new economic plan, Abenomics, to pump the economy. The plan initially helped in devaluating the currency, leading to a boom in export with a direct contribution to the economy.

The injection also pushed local investors into the stock market that saw a jump. However, in 2014, when things were running smooth, the government introduced taxes on expenditure to generate further cash inflow. The decision led to a recession. The negative impact of Abenomics continued until the end of 2017 when the economy started to see positive economic impact as per the GDP and domestic demand indication.

Hence, the use of Bitcoin as a diversifier for Nikkei 225 would not be effective unless the economic situation stabilizes. This is evidenced from the more recent 2021 and 2022 period as well. The green regions indicate the heightening correlation amidst the post-COVID inflation cum recession further sparked by the Russia-Ukraine war.

**Fig. 8:**
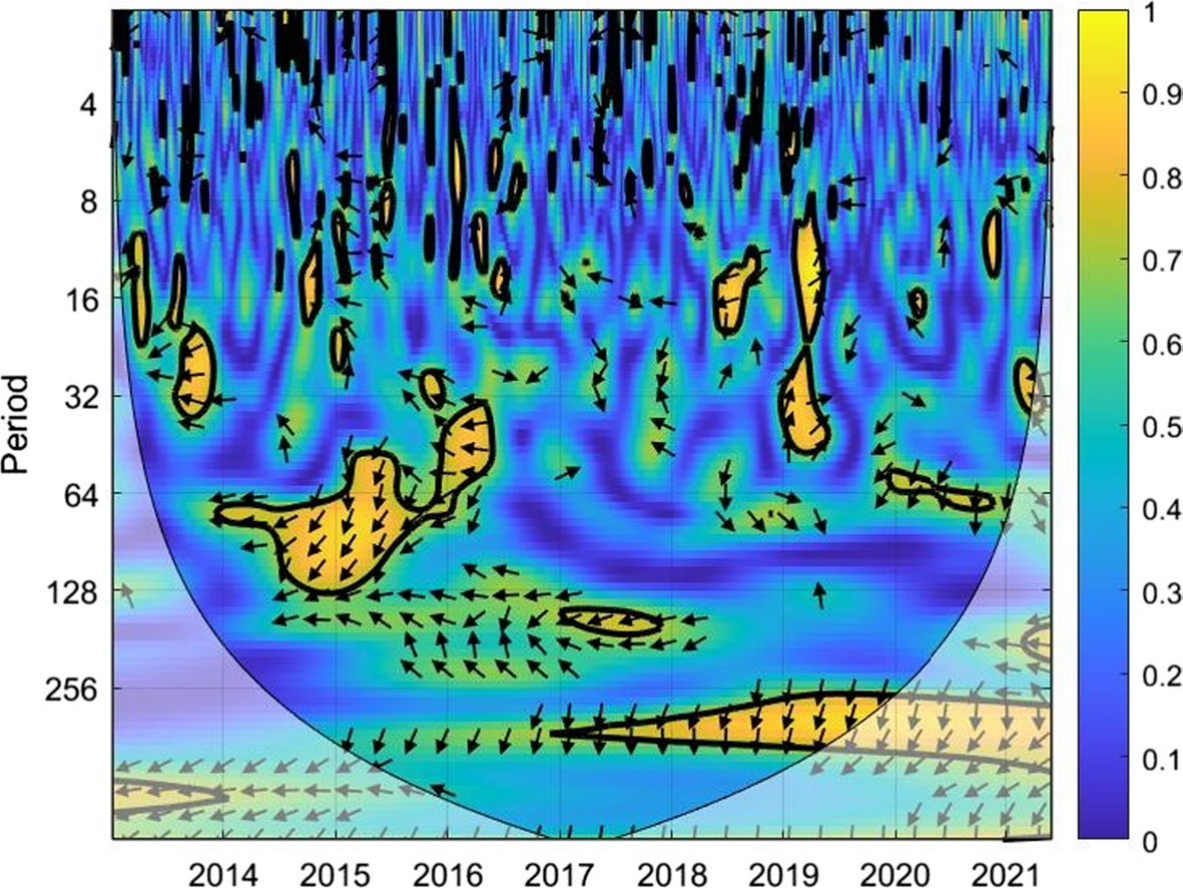
Wavelet coherence between gold and NIKKEI

As for the conditional correlation between gold and Nikkei returns, Fig. 8 illustrates that the pair works best for investment plans up to eight days, whereas mostly deep green and yellow marks make it less helpful to diversify for medium-term and long-term horizons.

For long-term investment plans, although high intercorrelation is observed between most indices globally during the COVID-19 pandemic, this particular pair manages to maintain a considerably low correlation during both the pandemic and the post-peak COVID-19 period. This suggests that gold could be a good fit for diversification in long term against the Nikkei 225 during future crises similar to the COVID-19 pandemic.

**Fig. 9:**
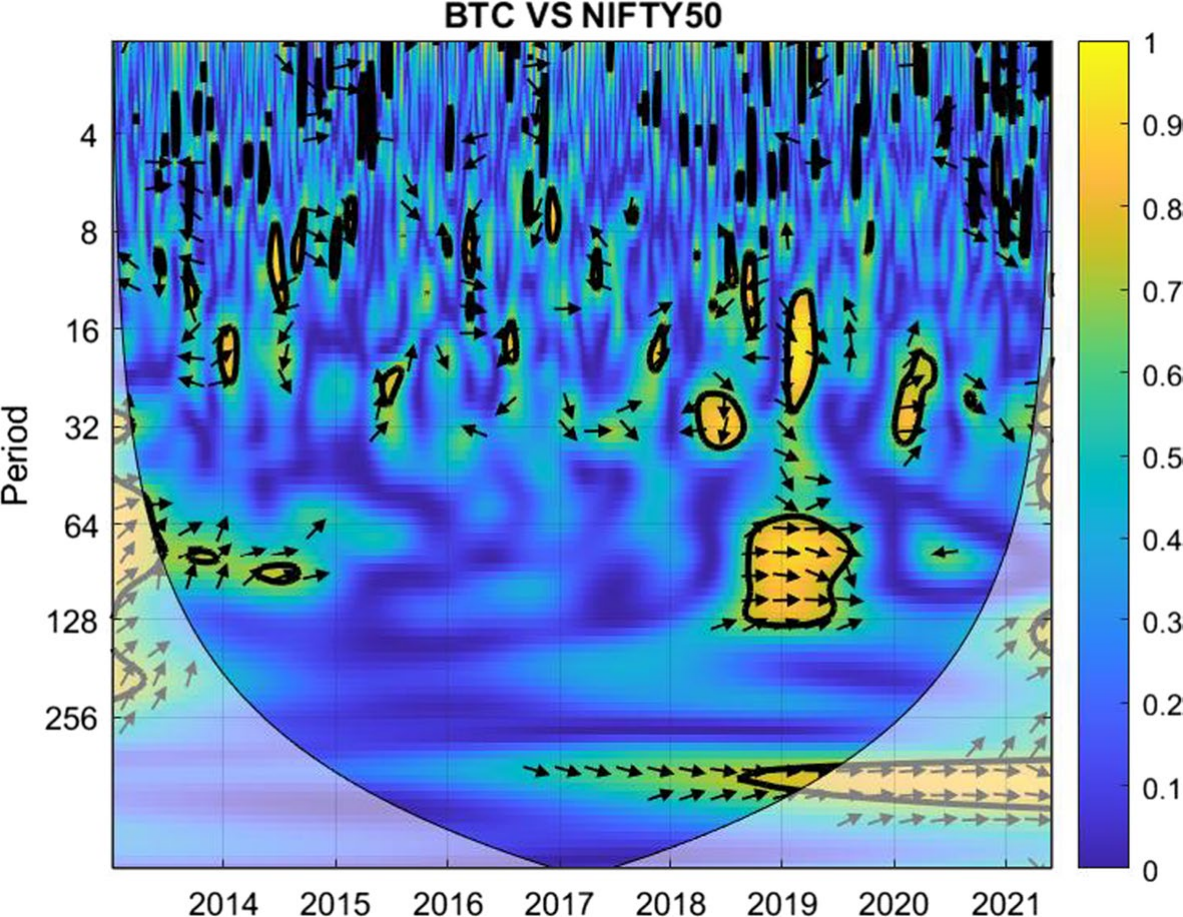
Wavelet coherence between Bitcoin and NIFTY 50

As shown in Fig. 9, Bitcoin and Nifty 50 work well for almost all time horizons at different levels, even during the recent Russia-Ukraine war.

For short-term investment, Bitcoin is weakly correlated with NIFTY 50 and offers effective portfolio diversification throughout the investigation period. Similar results hold for medium-term investment periods. The light blue and blue regions still suggest opportunities for diversification, except for the few yellow regions prior and during the COVID-19 periods.

For long-term investment periods of 64 days and above, the regions are dominated by blue within the thick contour, only marginally with green spots for 64–128 days in 2014 & 2015 and the notable yellow mark in late 2019 & early 2020 during the first stage of the COVID-19 pandemic. In particular, the consistently low correlation between the pair makes it ideal for investors looking for horizons of 128–256 days to consider.

**Fig. 10:**
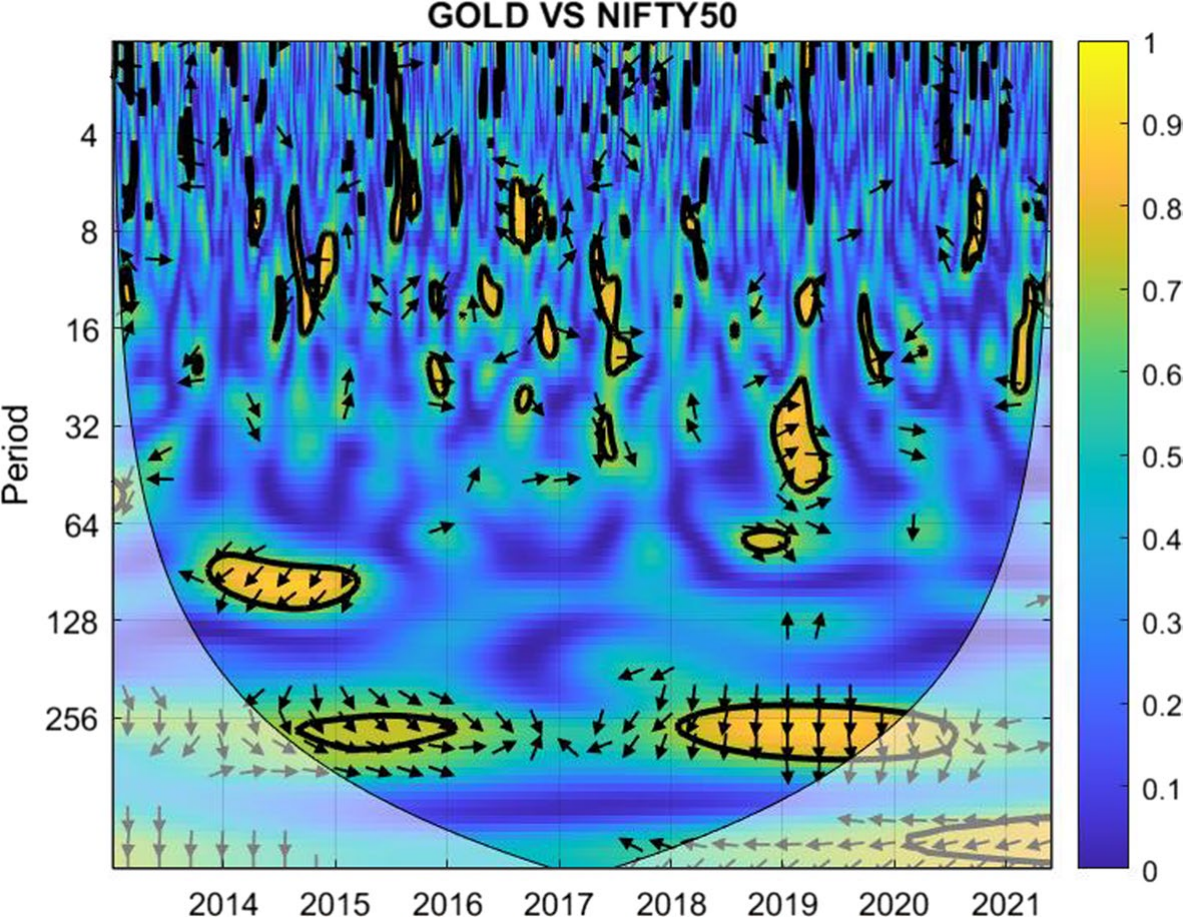
Wavelet coherence between gold and NIFTY 50

As shown in Fig. 10, unlike the case between Bitcoin and Nifty 50, the correlation between gold and Nifty 50 exhibits more yellow marks, except for short-term investment horizons up to four days. The use of gold to diversify the Indian stock market is not an effective choice, particularly for medium-term investment horizons.

**Fig. 11:**
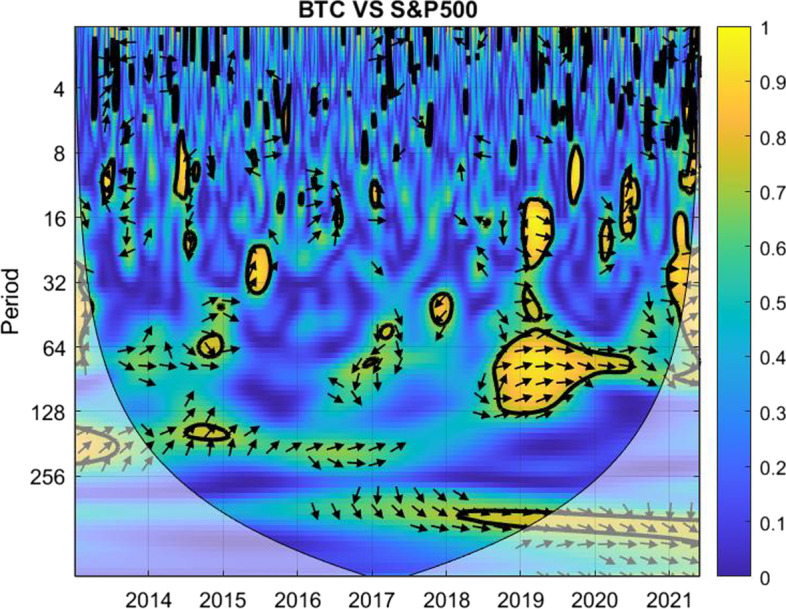
Wavelet coherence between Bitcoin and S&P 500

Between Bitcoin and S&P 500, Fig. 11 illustrates possibilities of diversification in short-term investment horizons. Although there are some green and yellow marks, the correlation vanishes shortly, making Bitcoin helpful to offset possible loss against S&P 500.

The medium-term investment performed relatively well as well until the global financial markets were hit by the COVID-19 pandemic. There are only very few green marks for investment periods ranging in 16–32 days until the first quarter of 2020. Following the first strike of COVID-19, the color turns from yellow to blue and from blue to green along with the subsequent variants. For investment periods ranging in 32–64 days, the main blue region is only spotted in 2016 and 2017, with the effect of the China-US trade war and the COVID-19 pandemic in other years.

Surprisingly, the correlation between the pair for investors planning for 128–256 days during the extreme crisis remains relatively balanced with more blue color, except in the first two years. This is generally not the case for investment plans ranging in 64–128 days though.

**Fig. 12:**
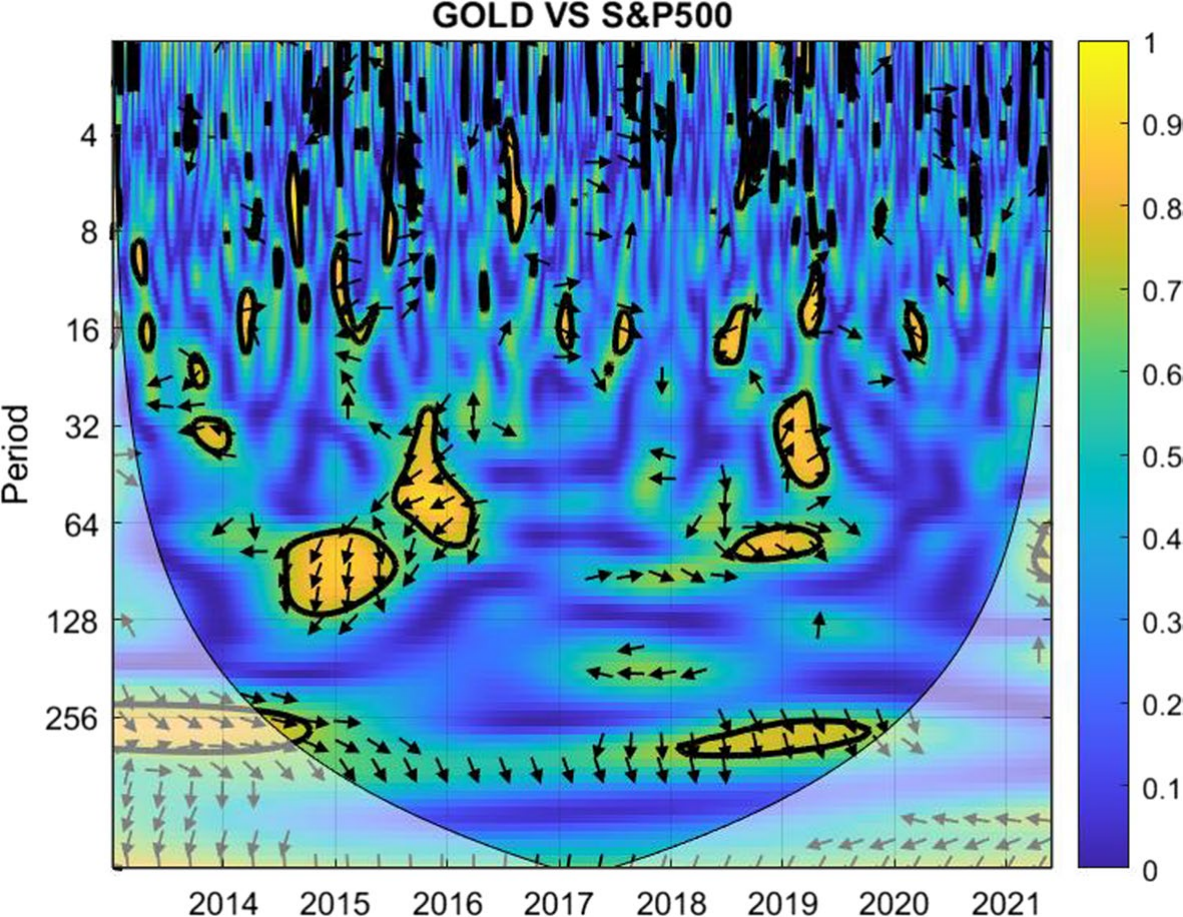
Wavelet coherence between gold and S&P 500

As shown in Fig. 12, irrespective of the financial and economic market condition, gold and the S&P 500 index work best for short-term investment horizons, as mostly blue to light blue regions are displayed with negligible yellow marks, which indicates diversification opportunities for investors.

For medium-term and long-term investment horizons, particularly for investment periods in the range of 32–128 days, notable yellow marks are observed. First, they are observed in 2015 to 2016 when the world saw a major fall in the oil price and the Greek debt default crisis, as well as the turbulence in the Chinese stock market, and then in late 2019 and early 2020 at the early stage of the COVID-19 pandemic.

The pair shields themselves from high correlation during the peak COVID-19 crisis coming with the subsequent variants of the Corona virus, making them a suitable choice for diversification.

**Fig. 13:**
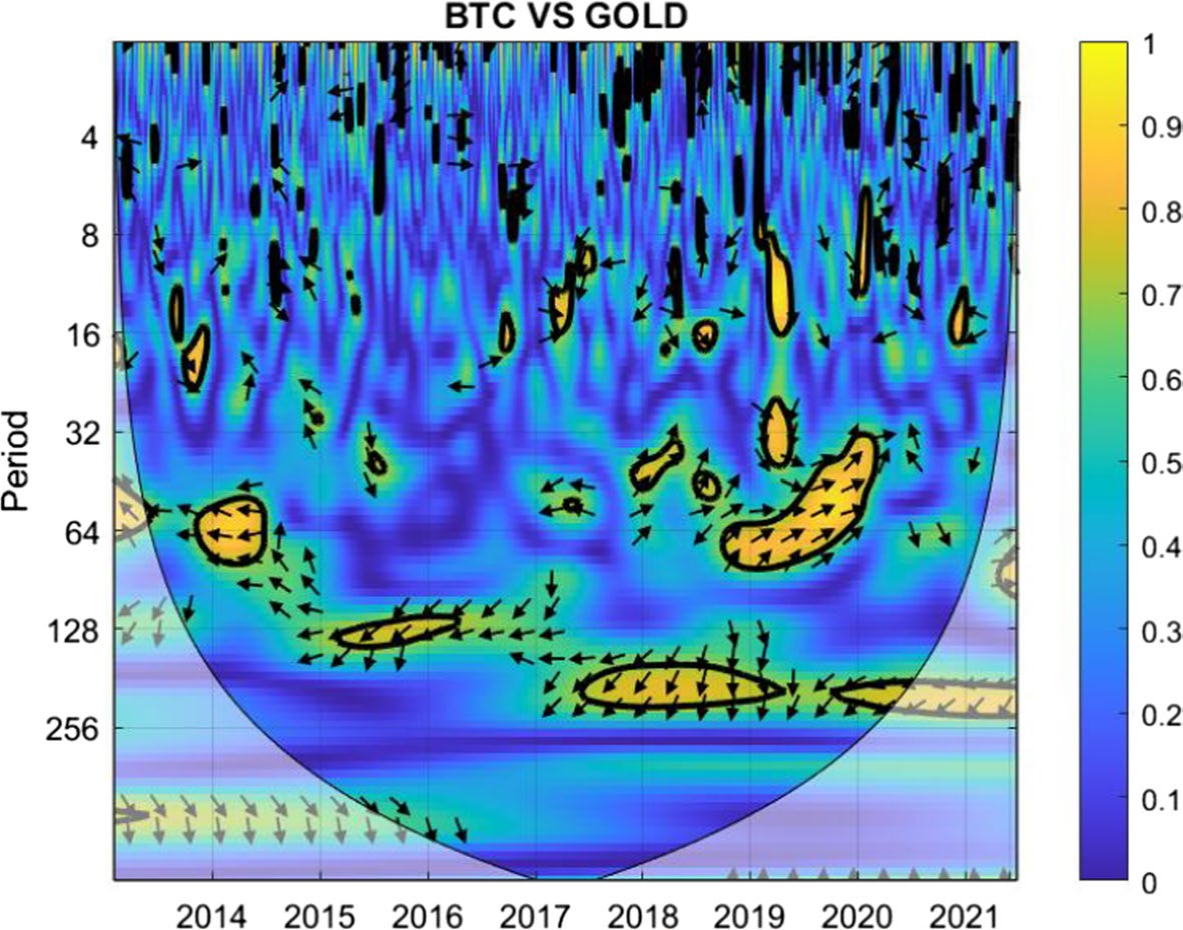
Wavelet coherence between Bitcoin and gold

For the correlation between Bitcoin and gold, Fig. 13 indicates possibilities for diversification up to 32 days. Relatively high correlation is observed for longer horizons, particularly during the crises. For example, the Bitcoin price increased following the outbreak of COVID-19, reassuring the thought of Bitcoin being a safe haven as gold, as evidenced by the yellow marks in the early days of COVID-19.

Hence, the analysis suggests that investors who hold a long-term plan and think of incorporating both Bitcoin and gold in the same portfolio should consider either one of them. Conversely, investors who look for investing in both could form two portfolios with possible different horizons, with Bitcoin in one and gold in the other.

Table 3 summarizes the wavelet coherence between Bitcoin, gold, and the indices.

**Table 3:** Summary of wavelet coherence of Bitcoin and gold with indices

		FTSE 100	FTSEINDO	FTSEMY	NIKKEI	NIFTY	S&P 500	GOLD
BITCOIN	Short term	$\surd$	$\surd$	$\surd$	$\surd$	$\surd$	$\surd$	$\surd$
	Medium term	$\surd ^{1}$	$\surd ^{2}$	$\times$	$\surd ^{3}$	$\surd ^{4}$	$\surd ^{5}$	$\surd ^{6}$
	Long term	$\surd ^{7}$	$\surd ^{8}$	$\surd ^{9}$	$\times$	$\surd ^{10}$	$\surd ^{11}$	$\times$
GOLD	Short term	$\surd$	$\surd$	$\surd$	$\surd$	$\surd ^{12}$	$\surd$	
	Medium term	$\times$	$\surd ^{13}$	$\surd ^{14}$	$\times$	$\times$	$\surd ^{15}$	
	Long term	$\surd ^{16}$	$\surd ^{17}$	$\surd ^{18}$	$\surd ^{19}$	$\times$	$\surd ^{20}$	

$$\surd$$: Diversification possible

$\times$: Diversification not possible

$^{1}$ Only possible during normal market condition

$^{2}$ Diversification possible as crisis prolongs

$^{3}$ Only possible during normal market condition

$^{4}$ Only possible for investment horizons of 32–64 days

$^{5}$ Possible during normal times for investment horizons of 32–64 days

$^{6}$ Possible during normal times for investment horizons within 32 days

$^{7}$ Only possible during normal market condition

$^{8}$ Diversification possible as crisis prolongs

$^{9}$ Diversification possible at least six months after the start of crisis

$^{10}$ Only possible during normal market condition

$^{11}$ Only possible for investment horizons of 128–256 days

$^{12}$ Possible during normal times for investment horizons within 4 days

$^{13}$ Only possible during normal market condition

$^{14}$ Only possible during normal market condition

$^{15}$ Only possible during normal market condition

$^{16}$ Only possible during normal market and crisis recovery conditions

$^{17}$ Only possible during normal market condition

$^{18}$ Only possible during normal market condition

$^{19}$ Only possible for investment horizons of 128–256 days

$^{20}$ Only possible during normal market condition

The analysis through wavelet coherence reveals how Bitcoin and gold correlate with the indices in different investment horizons, which is further related to and interpreted with the global and regional crises taking place during the period examined.

As summarized in Table 3, the wavelet results indicate that Bitcoin has low correlation with all the indices for short-term investment horizons in all market conditions, whereas possible conditional diversification evidence is also indicated for medium- and long-term investment horizons, particularly for investment periods up to 32 days or of 128–256 days in the normal market condition.

Considering the recent COVID-19 pandemic, Bitcoin is effective when paired with the three developing indices, i.e., FTSEINDO, FTSEMY, and NIFTY, in mitigating the relatively higher correlation, compared with the three developed counterparts, with which more yellow marks are exhibited, indicating even higher correlation.

Similar to the case of Bitcoin, the possible diversification offered by gold against the indices analyzed is time conditional when the market is in a stable condition or when a crisis enters into a more matured stage with volatility starting to turn down. Gold is effective with the developing indices than the developed indices.

Compared with Bitcoin, gold could be relatively lagging in the performance. For instance, gold is unable to offer diversification benefit for the medium-term investors in FTSE 100, Nikkei, and Nifty, as well as for long-term investors in Nifty.

#### Conditional volatility and correlation

For a complementary visual illustration of what behind the coherence exhibited from Figs. 1, 2, 3, 4, 5, 6, 7, 8, 9, 10, 11, 12 and 13 and outlined in Table 3, the estimated conditional volatilities and correlations of Bitcoin, gold, and the market indices from the multivariate GARCH DCC method discussed in the Multivariate GARCH section are displayed from Figs. 14, 15, 16 and 17, respectively.

**Fig. 14:**
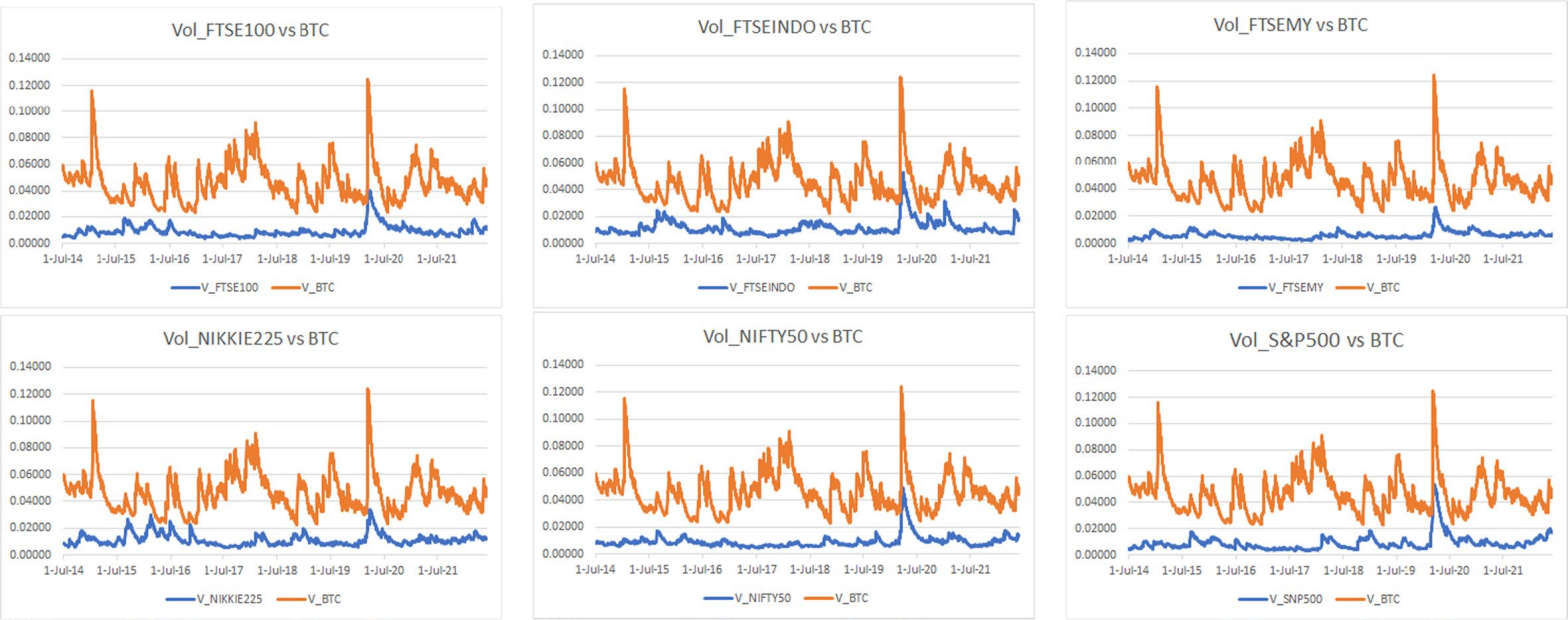
Estimated conditional volatilities of Bitcoin and indices

Figure 14 compares the level of volatility between Bitcoin and the indices. It can be observed that Bitcoin leads the run being the most volatile, whereas the rest follow each other with a stable trend. There is no doubt that all indices experienced significant volatility during the first quarter of 2020 owing to the announcement of the COVID-19 pandemic that introduced additional uncertain to the global markets. Despite the high volatility in general, Nikkei, FTSEMY, and gold are among the least sensitive in reacting to the COVID-19 crisis. Considering the recent Russia-Ukraine war, FTSEINDO, S&P 500, FTSE 100, and Nifty 50 are among the most sensitive in reacting to the war.

**Fig. 15:**
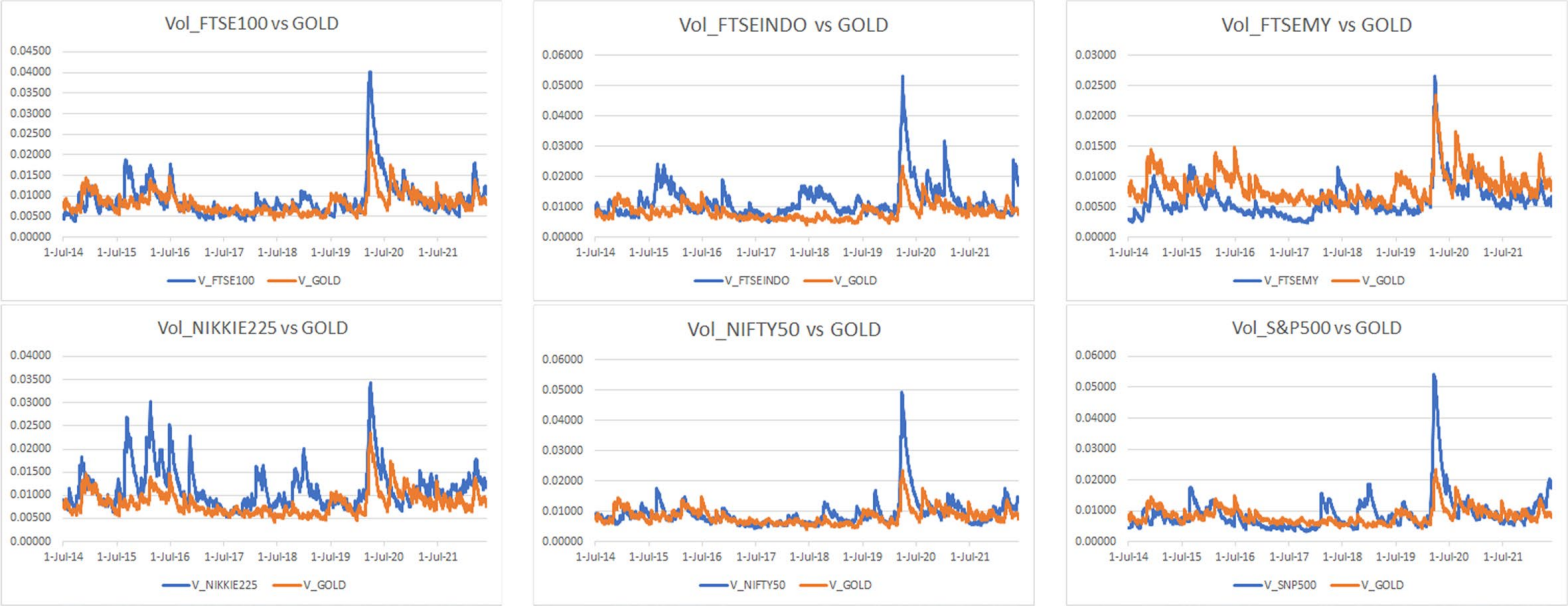
Estimated conditional volatilities of gold and indices

Considering the volatility level between gold and the indices, Fig. 15 shows that NIKKEI is the most volatile, whereas FTSEMY is the most stable and the other indices are approximately 60% more volatile on average during the COVID-19 pandemic. During the Russia-Ukraine war, the indices are roughly around 15% more volatile than gold, except for FTSEMY, which is approximately 33% less volatile while FTSEINDO is nearly 86% more volatile.

**Fig. 16:**
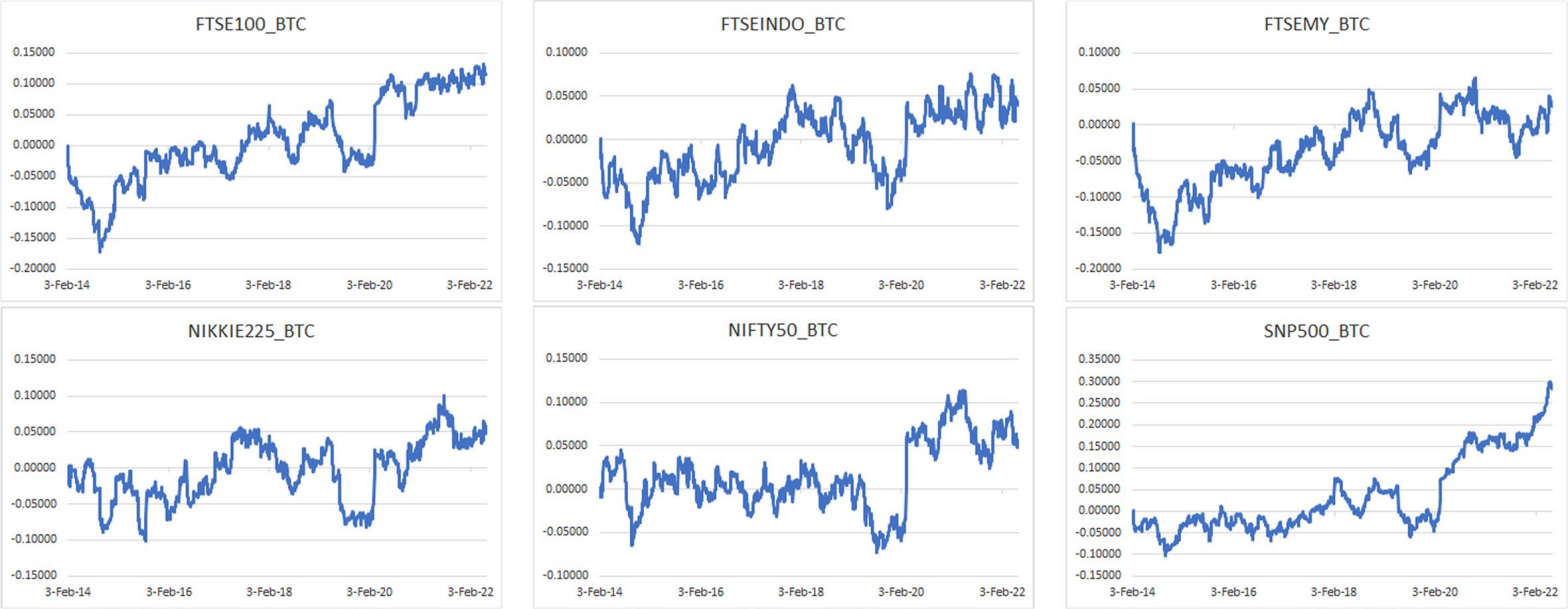
Estimated conditional correlations of Bitcoin and indices

Figure 16 illustrates the correlation between Bitcoin and the indices. Bitcoin is negatively correlated with all indices, except for NIFTY until 2017. The correlation then turns to be positive between Bitcoin and most indices in 2018 and then negative in 2019. Following the announcement of the COVID-19 pandemic, the correlation switches to positive, but with a significantly negligible magnitude, which is less than 0.01 in almost all instances except for S&P 500.

Generally, the pairwise correlation makes a surprising turn along with the announcement of the subsequent Delta and Omega variants of the Corona virus in 2021, starting to weaken, except for FTSE 100 and S&P 500. The correlation of the two pairs seems to rise with the pandemic as well as the more recent war between Russia and Ukraine.

Overall, during the early days of the COVID-19 crisis, all indices are positively but weakly correlated with Bitcoin. Diversification opportunities may still exist initially, particularly for investors looking for investing in the FTSEMY and gold, both of which pull back and come to a normal position right after the spike at the beginning of the sudden announcement.

**Fig. 17:**
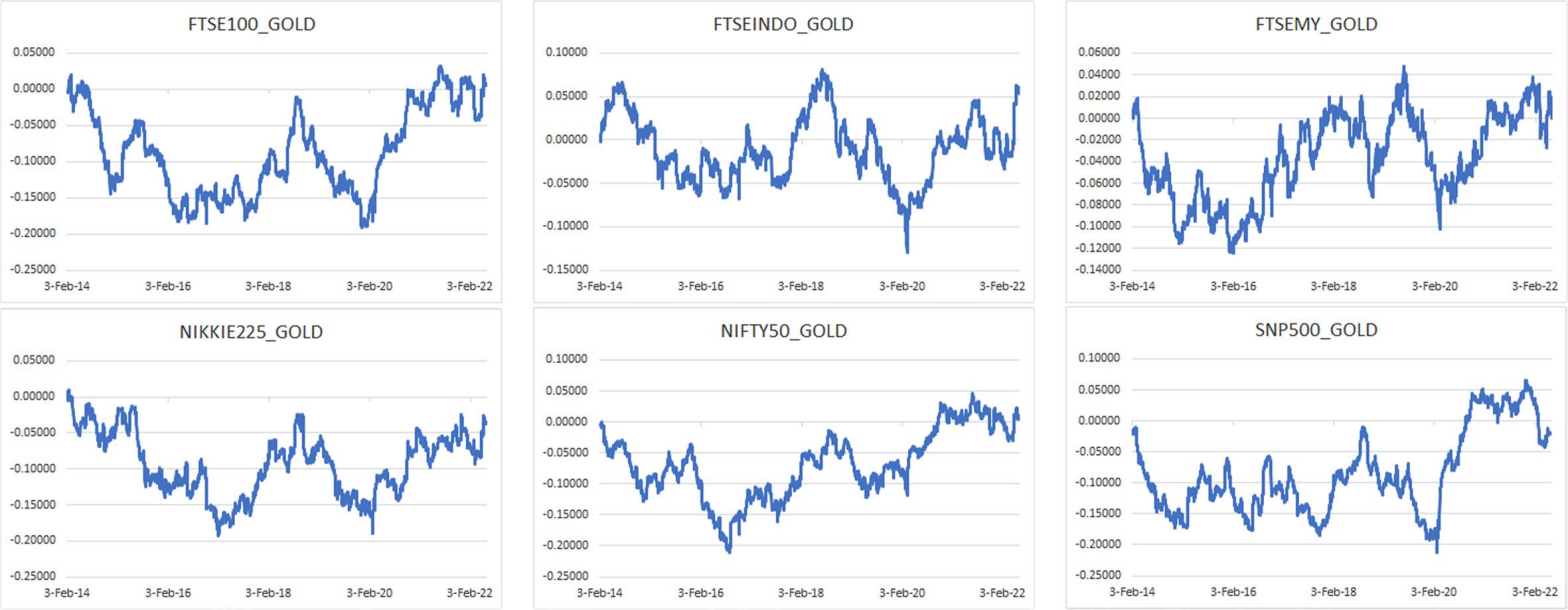
Estimated conditional correlations of gold and indices

For the correlation between gold and the indices, Fig. 17 shows a different scenario as in the case of Bitcoin. Gold is negatively correlated with almost all indices before the COVID-19 pandemic, with partial exception for FTSEINDO and FTSEMY. The correlation then turns to be in position with a steep upward slope following the announcement of the COVID-19 pandemic, except for Nikkei.

Concerning the latest war crisis, an upward trend in the correlation is also displayed for almost all indices. Despite the presence of positive correlation, the correlation is mostly statistically insignificant at the level below 0.1, allowing gold to work with the indices as a diversifier.

Figures 14 and 15 show that Bitcoin is the most volatile among all the sample indices considered, whereas gold is less exposed to volatility against the market indices. According to Figs. 16 and 17, Bitcoin is negatively correlated with almost all indices in normal periods, with FTSEMY being the most consistent in establishing negative correlation while NIFTY being the opposite. As for gold, it holds negative correlation with almost all indices as well during most of the time before the COVID-19 pandemic, with moderate exceptions for FTSEINDO.

The detailed estimated parameters of multivariate GARCH and unconditional volatilities & correlations are summarized in Tables 4 and 5, respectively, in Appendix. All *p*-values are statistically significant at the level of 5%.

#### Hedge ratio

For investors and fund managers, understanding the dynamics of hedge ratio at various time and frequency allows for efficient and effective portfolio fund allocation and eliminates possible mismanagement of resources. To further look into the hedging properties of Bitcoin and gold with the six indices, the hedge ratio is additionally examined (Antonakakis et al. 2018; Basher and Sadorsky 2016; Kroner and Ng 1998; Maghyereh and Abdoh 2022; Maghyereh et al. 2019).

To construct a hedge ratio with the DCC-GARCH model without loss of generality, it is assumed that a portfolio consists of a long position in Bitcoin or gold as asset *i* and a short position in one of the six indices as asset *j*. The variance of the portfolio $\textrm{Var}(r_{p,t})$ is then minimized by solving

$$\begin{aligned} \min _{\begin{array}{c} \beta _{ij, t} \end{array}} \textrm{Var}(r_{p, t}) = \min _{\begin{array}{c} \beta _{ij, t} \end{array}} \{\textrm{Var}(r_{i, t}) + \beta _t^2 \textrm{Var}(r_{j, t}) - 2 \beta _t \textrm{Cov}(r_{i, t}, r_{j, t})\}, \end{aligned}$$

which gives the time-varying optimal hedge ratio

$$\begin{aligned} \beta _{ij, t}^* = \frac{\textrm{Cov}(r_{i, t}, r_{j, t})}{\textrm{Var}(r_{i, t})}. \end{aligned}$$

The closer the $\beta _{ij, t}^*$ is to zero, the cheaper the financial asset is as a hedge for Bitcoin or gold, or vice versa.

**Fig. 18:**
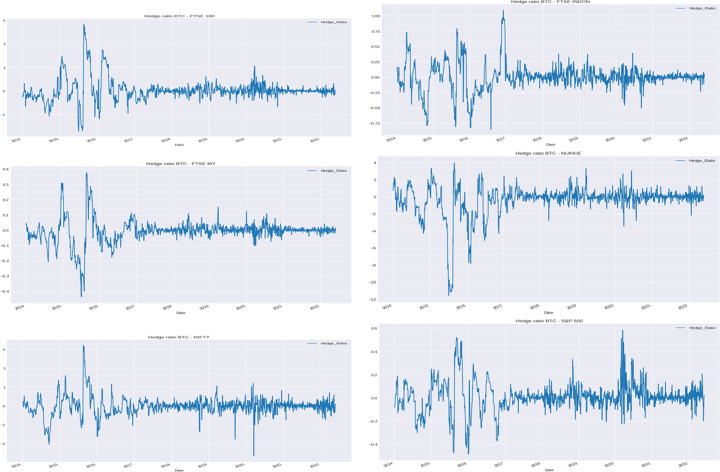
Time-varying hedge ratios of Bitcoin with indices

**Fig. 19:**
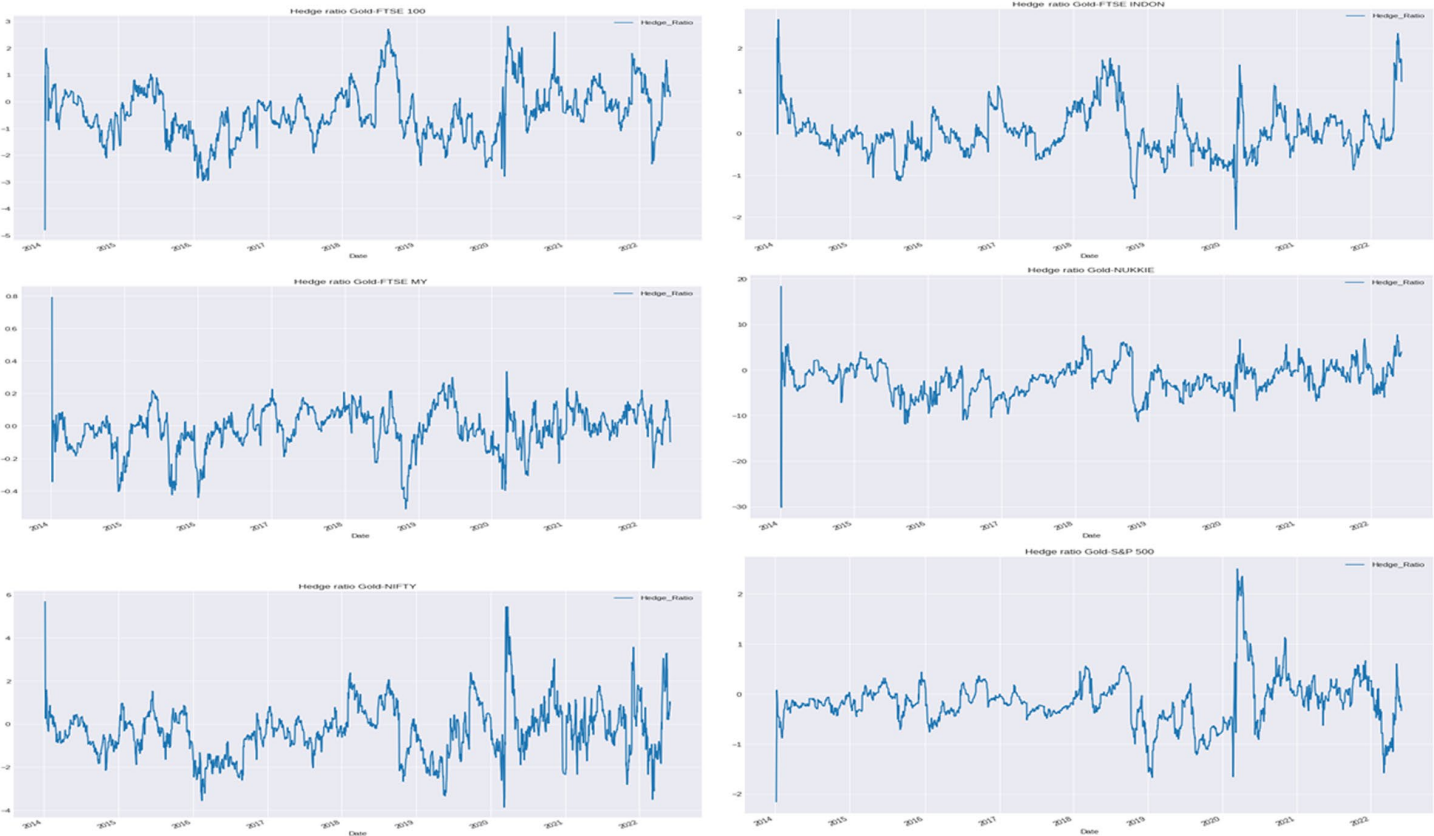
Time-varying hedge ratios of gold with indices

The time-varying hedge ratios of Bitcoin and gold with the indices are displayed in Figs. 18 and 19, respectively.

As shown in Fig. 18, although the hedge ratios differ over the sample period from 2014 to 2022, the hedge ratios are mostly close to zero. This indicates that Bitcoin is a cheap hedge for all six stock market indices. The modest exception is for the period from 2015 to 2016, during which the hedge ratios are considerably volatile. The relatively high volatility could be linked to the slowing GDP growth in China, drop in the oil price, June 2015 Greek debt default, effects of the end of US quantitative easing in October 2014, sharp rise in bond yields in early 2016, and June 2016 global equity market crash. After 2017, the hedge ratio almost remains consistent at the same level, except for the slight variability during 2020 due to the COVID-19 pandemic. Investors may consider investing in equity indices along with Bitcoin to diversify the COVID-19 pandemic as well as the Russia-Ukraine war risk. The hedge ratio shows that Bitcoin provides a reasonably less costly hedge with both emerging and developed equity markets.

As shown in Fig. 19, the hedge ratios of gold with FTSE100, Nikkei225, and Nifty50 exhibit high variability during the sample period from 2014 to 2022, commonly with peaks during the COVID-19 pandemic and unusually at the start of the sample period as well. The hedge ratios between gold and FTSE Indonesia, FTSE Malaysia, and S&P500 illustrate a significant different movement from the other three pairs, which interestingly suggests that gold may offer better hedging capability with the Indonesia, Malaysia, and US equity market indices, except during the COVID-19 pandemic and the Russia-Ukraine war.

The findings indicate that investors may think about investing in equity indices along with Bitcoin to diversify the risk of the COVID-19 pandemic and the Russia-Ukraine war given the potential losses from having volatile assets during the crises. Moreover, compared with gold, Bitcoin offers a better and more affordable means of hedging against the stock markets. The findings that Bitcoin serves as a reliable hedge for both emerging and developed equity markets are consistent with the similar outcomes revealed for the US stock and bond markets (Maghyereh and Abdoh 2022).

## Conclusion

Curiosity among investors about cryptocurrencies, particularly Bitcoin, has been increasing in the last decade. Although Bitcoin has attracted increasing attention from investors, its comparative diversification possibilities remain unclear under both normal and turbulent market conditions. This study tests the time-varying diversification ability of Bitcoin against different regional stock markets with greater emphasis on the recent COVID-19 pandemic and the ongoing Russia-Ukraine war to examine whether the prominent cryptocurrency offers similar diversification benefit as gold, which is conventionally considered as a safe heaven.

Using the daily data from January 7, 2014 to May 31, 2022 of FTSE 100, FTSE Indonesia as FTSEINDO, FTSE Bursa Malaysia KLCI as FTSEMY, NIKKEI, NIFTY, and S&P 500, along with Bitcoin and gold, this study employs the CWT and DCC through the multivariate GARCH model to test the diversification possibilities of Bitcoin and gold against the six representative stock markets during the pre-COVID period, COVID-19 pandemic, and post-COVID war crisis. The study aims to assess how the two behave against each other and if the former exhibits identical diversification benefit as possessed by the latter.

Among the six indices examined, FTSEINDO and FTSEMY have been barely considered in the literature. This study may be the first to test the time-varying diversification of Bitcoin and gold against more represented global stock indices during the period covering both the COVID-19 crisis and the post-COVID war crisis.

Overall, it is found that compared with gold, Bitcoin provides relatively better diversification opportunities during crises, which complements the findings of recent related studies (Jeribi et al. 2022; Mroua et al. 2022). The two work comparably in a normal market condition, which is consistent with findings from considering respective portfolios (Damianov and Elsayed 2020; Bouri et al. 2018).

It is demonstrated through CWT that both Bitcoin and gold fit well for diversifying short-term investment. For investment with longer periods, the conclusion is conditional. For instance, Bitcoin may only provide diversification during normal market conditions for FTSE 100 and Nikkei for all medium-term investment, and conditional for Nifty for investment horizons of 32–64 days. For investors in FTSEINDO looking for both the medium-term and long-term horizons, better diversification opportunity can be sought as the crisis period prolongs. For gold, medium-term diversification is mainly evidenced for FTSEINDO, FTSEMY, and S&P 500 only, and not for the other three indices.

For long-term investors, Bitcoin can be a better choice merely during normal times for FTSE 100 and Nifty, whereas gold could be a better fit in FTSE 100, FTSEINDO, FTSEMY, and S&P 500. Considering conditional investment, Bitcoin may offer diversification benefit for Malaysian investors six months after the pass of the crisis. S&P 500 investors may benefit from diversification with Bitcoin for investment periods of 128–256 days, similarly for investors in Nikkei for gold. Almost no room for diversification is found in long run for the pairs of Bitcoin and gold, Bitcoin and Nikkei, and gold and Nikkei.

Based on the volatility analysis through DCC, it is concluded that Bitcoin is significantly more volatile than gold and the indices considered, even under the normal market conditions. This may be one of the main remaining concerns for investors in Bitcoin. For gold, it possesses a more stable nature through the period assessed, except for the sudden shock induced by the COVID-19 pandemic. Despite having a spike during the early days of the pandemic, gold is less volatile compared with all indices, except for FTSEMY.

Considering the correlation of Bitcoin with the indices, a nearly identical pattern is observed, except for FTSE 100 and S&P 500. Similar results hold for gold with the indices, except for S&P 500, specifically with the recent war crisis being considered. Statistically, the pairwise positive correlation is almost consistently insignificant at the level of 0.05, with the main exception being Bitcoin during the early days of COVID-19. Particularly when paired with S&P 500, the correlation keeps on the rise since the start of the pandemic, making the pair the most vulnerable during the crisis.

The results from this study remind investors and portfolio managers planning to incorporate Bitcoin into their portfolios as a diversification tool to be additionally aware of the global geopolitical conditions in considering their investment tools and horizons. The findings also alarm investors and portfolio managers to decouple Bitcoin and gold from a single portfolio. Given the difference between Bitcoin and gold as investment tools, policy makers may direct financial institutions with Bitcoin being included in their portfolios to diversify the crisis risk, particularly in the case that gold is not present in the same portfolios.

This study focuses on Bitcoin. As the cryptocurrency market is receiving more attention globally, it is then worth looking into other cryptocurrencies in addition to Bitcoin (Fang et al. 2022), particularly under the recent more changing market conditions (Sebastião and Godinho 2021). DeFi could also be additionally considered for including stable coins.

## Data Availability

The datasets used and/or analysed during the current study are available from the corresponding author on reasonable request.

## Appendix

**Table 4:** Parameter estimates for multivariate GARCH with underlying multivariate normal distribution

Parameter	Estimate	Standard error	*t*-ratio	*p*-value
$\lambda _\mathrm{{BITCOIN}}^1$	0.80870	0.017126	47.2210	0.000
$\lambda _\mathrm{{GOLD}}^1$	0.94776	0.013943	67.9733	0.000
$\lambda _\mathrm{{FTSE 100}}^1$	0.81444	0.024595	33.1147	0.000
$\lambda _\mathrm{{FTSEINDO}}^1$	0.85239	0.023417	36.4011	0.000
$\lambda _\mathrm{{FTSEMY}}^1$	0.91016	0.013795	65.9798	0.000
$\lambda _\mathrm{{NIKKEI}}^1$	0.84664	0.023582	35.9023	0.000
$\lambda _\mathrm{{NIFTY}}^1$	0.89319	0.014572	61.2951	0.000
$\lambda _\mathrm{{S \& P 500}}^1$	0.75997	0.020344	37.3566	0.000
$\lambda _\mathrm{{BITCOIN}}^2$	0.17843	0.015427	11.5661	0.000
$\lambda _\mathrm{{GOLD}}^2$	0.03606	0.007019	5.1378	0.000
$\lambda _\mathrm{{FTSE 100}}^2$	0.11129	0.013328	8.3502	0.000
$\lambda _\mathrm{{FTSEINDO}}^2$	0.09373	0.012722	7.3673	0.000
$\lambda _\mathrm{{FTSEMY}}^2$	0.06519	0.008096	8.0527	0.000
$\lambda _\mathrm{{NIKKEI}}^2$	0.11320	0.014772	7.6630	0.000
$\lambda _\mathrm{{NIFTY}}^2$	0.06992	0.008147	8.5828	0.000
$\lambda _\mathrm{{S \& P 500}}^2$	0.16812	0.013868	12.1227	0.000
$\delta ^1$	0.99200	0.001207	821.8579	0.000
$\delta ^2$	0.00384	0.000475	8.0746	0.000

**Table 5:** Estimated unconditional volatility matrix of Bitcoin and gold with six indices

	BITCOIN	GOLD	FTSE 100	FTSEINDO	FTSEMY	NIKKEI	NIFTY	S&P 500
BITCOIN	0.06058	0.00555	0.08800	0.01080	$-$0.00558	0.01241	0.05431	0.12787
GOLD	0.00555	0.00879	$-$0.03121	0.01748	$-$0.01790	$-$0.08364	$-$0.00113	0.02865
FTSE 100	0.08800	$-$0.03121	0.01022	0.24405	0.28753	0.33496	0.45910	0.54747
FTSEINDO	0.01080	0.01748	0.24405	0.01328	0.40190	0.23113	0.39637	0.19883
FTSEMY	$-$0.00558	$-$0.01790	0.28753	0.40190	0.00671	0.35842	0.38525	0.13719
NIKKEI	0.01241	$-$0.08364	0.33496	0.23113	0.35842	0.01242	0.34587	0.19902
NIFTY	0.05431	$-$0.00113	0.45910	0.39637	0.38525	0.34587	0.01055	0.31199
S&P 500	0.12787	0.02865	0.54747	0.19883	0.13719	0.19902	0.31199	0.01101

The diagonal elements correspond to the unconditional volatilities and the off-diagonal elements to the correlations

## Abbreviations

CWT: Continuous wavelet transform
DCC: Dynamic conditional correlation
GARCH: Generalized auto-regressive conditional heteroskedasticity
